# Current and future perspectives on the regulation and functions of miR-545 in cancer development

**DOI:** 10.1016/j.cpt.2023.09.001

**Published:** 2023-09-05

**Authors:** Jinze Shen, Xinming Su, Qurui Wang, Yufei Ke, Tianyu Zheng, Yunan Mao, Zehua Wang, Jingyin Dong, Shiwei Duan

**Affiliations:** Key Laboratory of Novel Targets and Drug Study for Neural Repair of Zhejiang Province, School of Medicine, Hangzhou City University, Hangzhou, Zhejiang 310015, China

**Keywords:** miR-545, Competing endogenous RNA, Cell behavior, Biomarker, Therapeutic drug, Immunotherapy

## Abstract

Micro ribonucleic acids (miRNAs) are a highly conserved class of single-stranded non-coding RNAs. Within the miR-545/374a cluster, miR-545 resides in the intron of the long non-coding RNA (lncRNA) *FTX* on Xq13.2. The precursor form, pre-miR-545, is cleaved to generate two mature miRNAs, miR-545-3p and miR-545-5p. Remarkably, these two miRNAs exhibit distinct aberrant expression patterns in different cancers; however, their expression in colorectal cancer remains controversial. Notably, miR-545-3p is affected by 15 circular RNAs (circRNAs) and 10 long non-coding RNAs (lncRNAs), and it targets 27 protein-coding genes (PCGs) that participate in the regulation of four signaling pathways. In contrast, miR-545-5p is regulated by one circRNA and five lncRNAs, it targets six PCGs and contributes to the regulation of one signaling pathway. Both miR-545-3p and miR-545-5p affect crucial cellular behaviors, including cell cycle, proliferation, apoptosis, epithelial-mesenchymal transition, invasion, and migration. Although low miR-545-3p expression is associated with poor prognosis in three cancer types, studies on miR-545-5p are yet to be reported. miR-545-3p operates within a diverse range of regulatory networks, thereby augmenting the efficacy of cancer chemotherapy, radiotherapy, and immunotherapy. Conversely, miR-545-5p enhances immunotherapy efficacy by inhibiting T-cell immunoglobulin and mucin-domain containing-3 (TIM-3) expression. In summary, miR-545 holds immense potential as a cancer biomarker and therapeutic target. The aberrant expression and regulatory mechanisms of miR-545 in cancer warrant further investigation.

## Highlights

• Abnormal microRNA (miR)-545 expression is observed in cells, tissues, and serum across different cancer types.
• miR-545 faces competitive inhibition from 12 circular RNAs and 11 long non-coding RNAs, regulating 40 downstream genes.
• miR-545 modulates five key pathways and various cellular biological behaviors.
• miR-545 substantially affects cancer cell sensitivity to radiation and chemotherapy.
• miR-545 modulates immune cell infiltration levels and shows implications in immunotherapy.

## Graphical abstract

**Figure:**
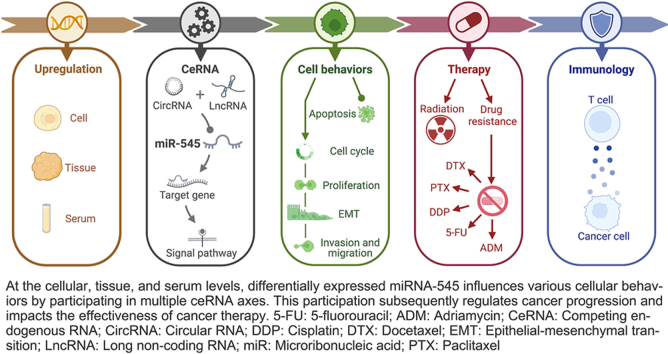

## Introduction

Micro ribonucleic acids (miRNAs) are endogenous single-stranded non-coding RNAs that are approximately 22 nucleotides long and are highly conserved in evolution.^1^ They weaken or eliminate the function of downstream protein-coding genes (PCGs) by binding to the 3′-untranslated regions (3′-UTR) of target gene mRNA and play a role in post-transcriptional regulation.^2^ The competing endogenous RNA (ceRNA) regulatory network connects long non-coding RNAs (lncRNAs), circular RNAs (circRNAs), miRNAs, and PCGs. miRNAs regulate cancer progression by participating in the ceRNA regulatory axis and affecting downstream PCG expression and signaling pathway activation.^3^

miR-545 is located at Xq13.2, within intron 1 of the lncRNA *FTX* (*FTX* Transcript, XIST Regulator), and it has been associated with cancers in multiple systems, including the nervous,^4^^,^^5^ respiratory,^6^^,^^7^ digestive^8^ and motor^9^ systems. miR-545 is intricately involved in an extensive ceRNA regulatory network, and it is competitively repressed by 15 circRNAs and 12 lncRNAs. It plays a crucial role in regulating multiple signaling pathways, including the catenin,^10^ phosphoinositide-3-kinase (PI3K)/protein kinase B (AKT),^8^^,^^11^ cell cycle,^12^, ^13^, ^14^ and p38 pathways.^15^ Via its participation in these pathways, miR-545 exerts control over critical cellular processes, such as cell cycle progression, proliferation, apoptosis, epithelial-mesenchymal transition (EMT), invasion, and migration.

This study examines miR-545 dysregulation in various human cancers and summarized the genes and pathways involved in its regulation and their roles *in vivo* and *in vitro*. Our work also provides a systematic summary of the association between miR-545 and patient prognosis as well as the relationship of miR-545 with various therapeutic approaches. Finally, we discuss the current research progress and limitations of miR-545 to provide directions for future translational medicine research.

## miR-545 expression dysregulation in human cancers

Pre-miR-545 is processed into two mature miRNA products: miR-545-3p and miR-545-5p. Both of these miRNAs are expressed in various human cell types. Research has shown that both miR-545-3p and miR-545-5p levels are abnormal in 14 different cancer types Supplementary Table 1. Specifically, miR-545-3p levels were lower than normal in 11 cancers and higher than normal in two cancers. In contrast, miR-545-5p levels were lower than normal in seven types of cancer.

As shown in Supplementary Table 2 and Supplementary Figure 1, based on miRNA data from The Cancer Genome Atlas (TCGA) database, we explored the abnormal expression of miR-545 in over 30 types of cancer and paracancerous tissues and its association with the host gene *FTX*. Our results showed that pre-miR-545 expression was markedly negatively correlated with *FTX* in three tumor types: kidney chromophobe (KICH), mesothelioma (MESO), and uterine carcinosarcoma (UCS). miR-545-3p expression was upregulated in uterine corpus endometrial carcinoma (UCEC) and markedly negatively correlated with *FTX i*n three tumor types: MESO, rectum adenocarcinoma (READ), and thymoma (THYM). miR-545-5p expression was upregulated in four tumors, head and neck squamous cell carcinoma, lung adenocarcinoma (LUAD), lung squamous cell carcinoma (LUSC), and UCEC, and substantially negatively correlated with *FTX* in four tumors: colon adenocarcinoma (COAD), KICH, LUAD, and prostate adenocarcinoma (PRAD) (see Supplementary Material for details).

Conflicting results have been reported regarding the expression patterns of miR-545-3p in colorectal cancer (CRC). Several studies utilizing quantitative reverse transcription polymerase chain reaction (RT-qPCR) have demonstrated that miR-545-3p expression is reduced in both CRC cell lines and tissues compared to that in normal colonic epithelium and non-tumor tissues, respectively.^11^^,^^16^^,^^17^ However, another study using microarray profiling found that miR-545-3p expression was higher in the serum exosomes of CRC patients than that in healthy individuals.^18^ The inconsistencies in miR-545-3p expression patterns in CRC may be attributed to differences in cancer tissues or measurement methods used in different studies.

In summary, variations exist in the expression of miR-545 across different contexts, and its discrepant expression in experimental data compared to that in the TCGA database has been debated. This disparity could potentially be attributed to the limited number of normal control samples in the TCGA database, leading to an imbalanced sample size in the cancer group. For instance, TCGA included 218 CESC tumor samples but only two control samples. Additionally, the reduced number of reads matching the gene in certain chips or algorithms may result from the lower accuracy of transcriptome expression results for low-expression genes as well as the short fragments and lower abundance of miR-545. Consequently, this can contribute to false results. Moreover, errors in measurement outcomes can stem from the biological properties of miRNAs. For instance, if miR-545 expression undergoes subtle changes in the early stages of cancer but either remains relatively stable during cancer progression or is subject to passive regulation by other genes within the gene regulatory network, it can lead to abnormal fluctuations in its expression levels. Therefore, further studies with larger sample sizes and diverse sample types are necessary to gain a more comprehensive understanding of the aberrant expression patterns of miR-545-3p and miR-545-5p in cancer.

## miR-545 and its host gene *FTX*

*FTX* is a well-studied lncRNA that plays a crucial role in X chromosome inactivation. Located upstream of the *XIST* gene in the X chromosome inactivation center (XIC), *FTX* can enhance *XIST* expression and trigger X chromosome inactivation.^19^ Additionally, *FTX* interacted with DExH-box helicase 9 (*DHX9*) and endoribonuclease dicer (*DICER)* to positively regulate both A-to-I RNA editing and miRNA expression.^11^

miR-374b/421 and miR-545/374a clusters are located within the *FTX* intron.^20^ Both *FTX* and miR-545-3p are upregulated and exhibit tumor-promoting functions in CRC.^11^ However, *FTX* suppressed miR-545-5p expression in monocytes from patients with liver cirrhosis.^21^ These findings suggest that regulation of miR-545-3p/5p expression by the lncRNA *FTX* may be tissue-specific and dependent on the host *FTX* transcript.

We analyzed TCGA data and found that pre-miR-545 was substantially negatively correlated with *FTX* in three tumor types (KICH, MESO, and UCS) but was substantially positively correlated with *FTX* in skin cutaneous melanoma. miR-545-5p expression was substantially negatively correlated with *FTX* levels in COAD, KICH, LUAD, and PRAD. miR-545-3p was substantially negatively correlated with *FTX* levels in the MESO, READ, and THYM groups. Additionally, the expression levels of pre-mir-374a, pre-mir-374b, pre-miR-374c, pre-mir-421, and pre-mir-545 in the miR-374b/421 and miR-545/374a clusters were strongly positively correlated Supplementary Material. This suggests that the regulation of the expression of miR-545 and that of its neighboring genes is related and may be tissue-specific.

As the host gene of miR-545, *FTX* is located on the X chromosome, it is worth paying attention to sex differences in its expression changes. TCGA data showed that sex differences in miR-545 expression were not significant in most cancers, as only four (pheochromocytoma and paraganglioma, thyroid carcinoma [THCA], MESO, and READ) showed significant differences (see Supplementary Material for details). Previous studies have not considered the sex of the cell line source. More in-depth research is needed to verify whether sex-related differences exist in the regulation of miR-545 expression.

## miR-545 and its competing endogenous ribonucleic acids (ceRNAs)

**Figure 1:**
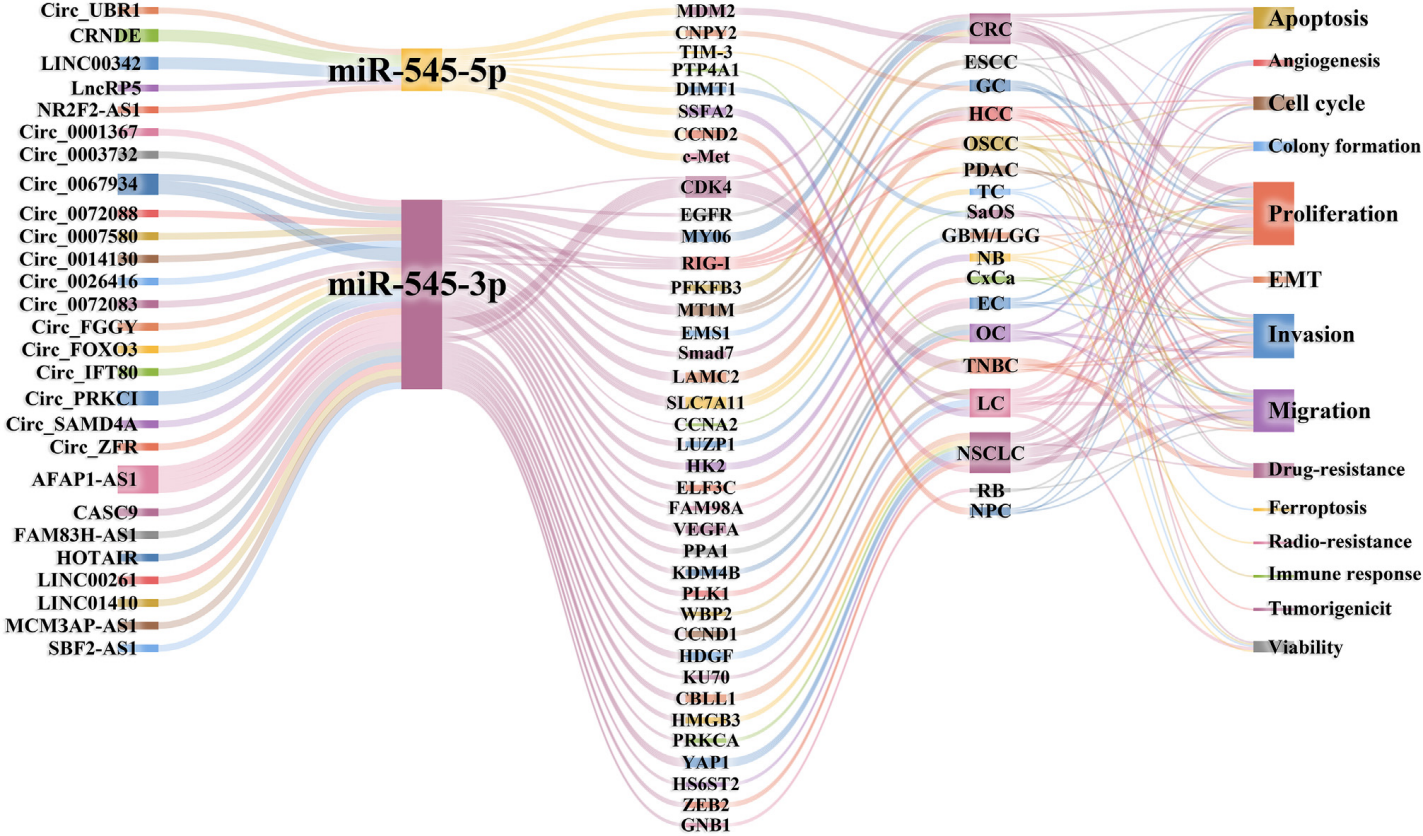
miR-545-related competing endogenous RNA (ceRNA) networks in cancer. The ceRNA network involving microRNAs included 27 ceRNAs and 39 PCGs that regulated cancer cell behavior in 18 cancers. AFAP1: Actin filament associated protein 1; AS1: Antisense RNA 1; CASC9: Cancer susceptibility candidate 9; CBLL1: Casitas B-lineage lymphoma-transforming sequence-like protein 1; CCNA2: Cyclin A2; CCND1: Cyclin D1; CCND2: Cyclin D2; CDK4: Cyclin-dependent kinase 4; Circ: Circular; c-Met: Mesenchymal-epithelial transition factor; CNPY: Canopy FGF signaling regulator; CRC: Colorectal cancer; CRNDE: Colorectal neoplasia differentially expressed; CxCa: Cervical carcinoma; DIMT1: DIM1 RRNA methyltransferase and ribosome maturation factor 1; EC: Endometrial cancer; EGFR: Epidermal growth factor receptor; ELF3C: E74 like ETS transcription factor 3C; ESM1: Endothelial cell specific molecule 1; EMT: Epithelial-mesenchymal transition; ESCC: Esophageal squamous cell carcinoma; FAM83H: Family with sequence similarity 83 member H; FAM98A: Family with sequence similarity 98 member A; FGGY: GGY carbohydrate kinase domain containing; FOXO3: Transcription factor forkhead box O-3; GBM/LGG: Glioblastoma/lower grade glioma; GC: Gastric cancer; GNB1: G protein subunit beta 1; HCC: Hepatocellular carcinoma; HDGF: Heparin binding growth factor; HK2: Hexokinase II; HMGB3: High-mobility group box 3; HOTAIR: HOX transcript antisense RNA; HS6ST2: Heparan sulfate 6-O-ulfotransferase 2; IFT: Intraflagellar transport; KDM3B: Lysine demethylase 3B; KU70: X-ray repair cross complementing 6; LAMC: Laminin subunit gamma 2; LC: Lung cancer; LINC: Long intergenic noncoding; Lnc: Long non-coding; LUZP1: Leucine zipper protein 1; MCM3AP: Minichromosome maintenance complex component 3 associated protein; MDM2: Murine double minute 2; miR: Micro ribonucleic acid; MT1M: Metallothionein 1M; MYO6: Myosin VI; NB: Neuroblastoma; NPC: Nasopharyngeal carcinoma; NRF2F2: Nuclear factor erythroid 2-related factor 2; NSCLC: Non-small cell lung cancer; OC: Ovarian cancer; OSCC: Oral squamous cell carcinoma; PDAC: Pancreatic ductal adenocarcinoma; PFKFB3: 6-phosphofructo-2-kinase/fructose-2,6-biphosphatase 3; PLK1: Polo like kinase 1; PPA1: Inorganic pyrophosphatase 1; PRKCA: Protein kinase c alpha; PRKCI: Protein kinase v Iota; PRP3A1: Protein tyrosine phosphatase type IVA 1; PTP4A1: Protein tyrosine phosphatase 4A1; RB: Retinoblastoma; RIG-I: Retinoic acid-inducible gene I; SAMD4A: Sterile alpha motif domain containing 4A; SaOS: Osteosarcoma; SBF2: SET binding factor 2; SLC7A11: Solute carrier family 7 member 11; Smad: Small mother against decapentaplegic; SSFA2: Sperm-specific antigen 2; TC: Thyroid carcinoma; TIM-3: T-cell immunoglobulin and mucin-domain containing-3; TNBC: Triple-negative breast cancer; UBR1I: Ubiquitin protein ligase E3 component N-Recognin 1; VEGFA: Vascular endothelial growth factor A; WBP2: WW domain binding protein 2; YAP1: Yes1 associated transcriptional regulator; ZEB2: Zinc finger E-box binding homeobox 2; ZFR: Zinc finger RNA binding protein.

CeRNAs competitively bind to miRNA, weakening its inhibitory effect on target mRNA and regulating cellular activities at the post-transcriptional level.^22^ As shown in Supplementary Table 3 and Figure 1, the ceRNA regulatory network of miR-545 involves circRNAs and lncRNAs that play regulatory roles in cell biology.

### Micro ribonucleic acid-545–related circular ribonucleic acids (CircRNAs)

CircRNAs are non-coding RNA with closed circular structures that can bind to miRNAs to regulate downstream gene expression.^23^ As shown in Supplementary Table 3 and Figure 1, the inhibitory effects of miR-545 on target genes were competitively inhibited by 15 circRNAs: Circ_PRKCI, Circ_0067934, Circ_0072083, Circ_0026416, Circ_0067835, Circ_0003732, Circ_0001367, Circ_FOXO3, Circ_UBR1, Circ_0007580, Circ_0014130, Circ_0072008, Circ_FGGY, Circ_ZFR, and Circ_SAMD4A.

Nineteen circRNA/miR-545-3p axes promote cancer progression, including Circ_PRKCI/miR-545-3p/E2F7 in glioblastoma (GBM)/low-grade glioma (LGG)^24^; Circ_0072083/miR-545-3p/CBLL1 in lung cancer (LC)^7^; Circ_0007580/miR-545-3p/PRKCA,^15^ Circ_FOXO3/miR-545-3p/HMGB3,^25^ Circ_0014130/miR-545-3p/YAP1,^26^ Circ_PRKCI/miR-545-3p/E2F7,^27^ and Circ_PRKCI/miR-545-3p in gastric cancer (GC)^8^; Circ_FGGY/miR-545-3p/SMAD7 in HCC^28^; Circ_0026416/miR-545-3p/MYO6 in CRC^16^; Circ_SAMD4A/miR-545-3p/PFKFB3^29^ and Circ_0003732/miR-545-3p/CCNA2 in osteosarcoma (SaOS)^30^; Circ_0072008/miR-545-3p/SLC7A11 in pancreatic ductal adenocarcinoma (PDAC)^60^; Circ_IFT80 (Circ_0067835)/miR-545-3p/FAM98A in endometrial cancer (EC)^31^; Circ_0067934/miR-545-3p/PPA1 in ovarian cancer (OC)^32^; Circ_PRKCI/miR-545-3p/WBP2^33^ and Circ_0067934/miR-545-3p/EIF3C in cervical carcinoma (CxCa)^34^; Circ_ZFR/miR-545-3p/WMT5A in bladder cancer; and Circ_0067934/miR-545-3p/SLC7A11 axis in THCA.^35^ Additionally, the Circ_UBR1/miR-545-5p/SSFA2 axis promoted the malignant behavior of cancer cells in LC.^36^ However, the Circ_0001367/miR-545-3p/LUZP1 axis suppressed cancer progression in GBM/LGG.^4^

### miR-545–related long non-coding ribonucleic acids (lncRNAs)

LncRNAs are long non-coding RNAs longer than 200 nt.^37^ As shown in Supplementary Table 3 and Figure 1, 12 lncRNAs competitively inhibit miR-545: AFAP1-AS1, CRNDE, HOTAIR, LINC00342, SBF2-AS1, CASC9, LINC00261, MCM3AP-AS1, LINC01410, LncRP5, FAM83H-AS1, and NR2F2-AS1.

Nine lncRNA/miR-545-3p/PCG axes promote cancer progression, including AFAP1-AS1/miR-545-3p/HDGF^6^ and FAM83H-AS1/miR-545-3p/HS6ST2^38^ in LC; CASC9/miR-545-3p/LAMC2 in oral squamous cell carcinoma (OSCC)^39^; SBF2-AS1/miR-545-3p/EMS1 in GC^40^; HOTAIR/miR-545-3p/EGFR^17^ and MCM3AP-AS1/miR-545-3p/CDK4^12^ in CRC; AFAP1-AS1/miR-545-3p/VEGFA in EC^41^; AFAP1-AS1/miR-545-3p/CDK4 in triple-negative breast cancer (TNBC),^13^ and AFAP1-AS1/miR-545-3p/GNB1 in retinoblastoma (RB).^42^ Two lncRNA/miR-545-3p/PCG axes can suppress cancer progression, including the LINC01410/miR-545-3p/HK2 axis in neuroblastoma (NB) to suppress tumorigenesis and tumor radioresistance^5^ and the LINC00261/miR-545-3p/MT1M axis to suppress cisplatin resistance in esophageal squamous cell carcinoma (ESCC) cell lines.^43^

Five lncRNA/miR-545-5p/PCG axes promote cancer progression, including CRNDE/miR-545-5p/CCND2 in nasopharyngeal carcinoma (NPC),^44^ NR2F2-AS1/miR-545-5p/MET in LC,^45^ LncRP5/miR-545-5p/PTP4A1 in OC,^46^ CRNDE/miR-545-5p/TIM-3 in OSCC,^47^ and LINC00342/miR-545-5p/MDM2 in CRC.^48^

## miR-545 regulates cell behaviors

**Figure 2:**
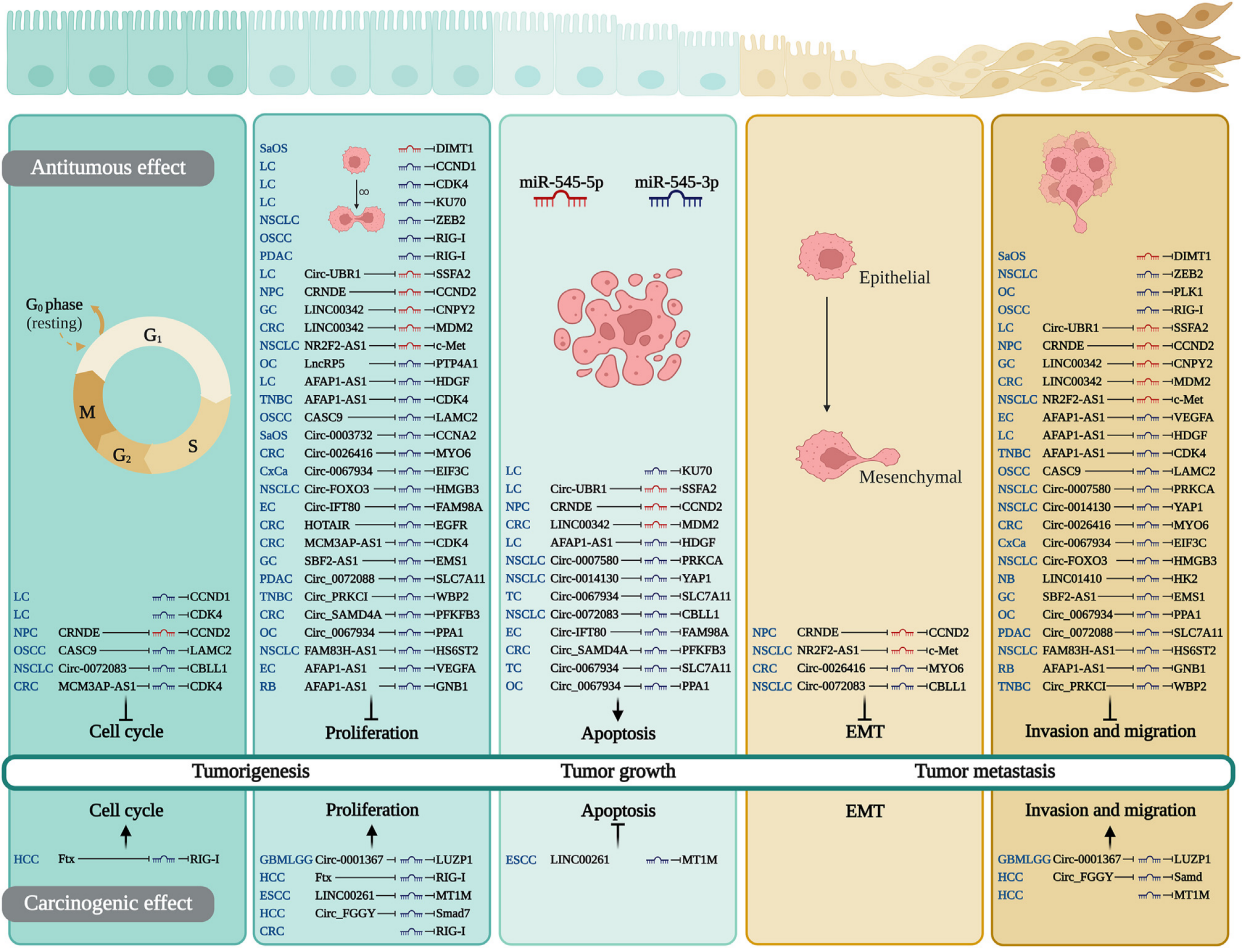
Elucidating the mechanism of miR-545 in regulating cancer cell behavior. miR-545 can regulate the behaviors of various cancer cells by participating in a competing endogenous RNA network or by targeting protein-coding genes. AFAP1: Actin filament associated protein 1; AS1: Antisense RNA 1; CASC9: Cancer susceptibility candidate 9; CBLL1: Casitas B-lineage lymphoma-transforming sequence-like protein 1; CCND: Cyclin D; CDK4: Cyclin-dependent kinase 4; Circ: Circular; c-Met: Mesenchymal-epithelial transition factor; CNPY: Canopy FGF signaling regulator; CRC: Colorectal cancer; CRNDE: Colorectal neoplasia differentially expressed; CxCa: Cervical carcinoma; EC: Endometrial cancer; EGFR: Epidermal growth factor receptor; EIF3C: Eukaryotic translation initiation factor 3 subunit C; EMS1: Endothelial cell specific molecule 1; EMT: Epithelial-mesenchymal transition; FAM38H: Family with sequence similarity 83 member H; FGGY: GGY carbohydrate kinase domain containing; GC: Gastric cancer; GNB1: G protein subunit beta 1; HDGF: Heparin binding growth factor; HMGB3: High-mobility group box 3; HOTAIR: HOX transcript antisense RNA; KU70: X-ray repair cross complementing 6; LAMC2: Laminin subunit gamma 2; LC: Lung cancer; LINC: Long intergenic noncoding; LUZP1: Leucine zipper protein 1; MCM3AP: Minichromosome maintenance complex component 3 associated protein; MDM2: Murine double minute 2; miR: Micro ribonucleic acid; MT1M: Metallothionein 1M; MYO6: Myosin VI; NB: Neuroblastoma; NPC: Nasopharyngeal carcinoma; NR2F2: Nuclear factor erythroid 2-related factor 2; NSCLC: Non-small cell lung cancer; OC: Ovarian cancer; OSCC: Oral squamous cell carcinoma; PDAC: Pancreatic ductal adenocarcinoma; PFKFB3: 6-phosphofructo-2-kinase/fructose-2,6-biphosphatase 3; PPA1: Inorganic pyrophosphatase 1; PRKCI: Protein kinase v Iota; PTP4A1: Protein tyrosine phosphatase 4A1; RB: Retinoblastoma; RIG-I: Retinoic acid-inducible gene I; SAMD4A: Sterile alpha motif domain containing 4A; SaOS: Osteosarcoma; SBF2: SET binding factor 2; Smad: small mother against decapentaplegic; SSFA2: Sperm-specific antigen 2; TC: Thyroid carcinoma; TNBC: Triple-negative breast cancer; UBR1: Ubiquitin protein ligase E3 component N-recognin 1; VEGFA: Vascular endothelial growth factor A; WBP2: WW domain binding protein 2; YAP1: Yes1 associated transcriptional regulator; ZEB-2: Zinc finger E-box binding homeobox 2.

Because miRNAs and their target gene sequences may not be fully complementary, miRNAs have a wide range of regulatory functions that affect cancer progression by regulating various biological behaviors.^49^ As shown in Supplementary Table 4 and Figure 2, multiple ceRNA/miR-545/PCG axes can regulate cell cycle, proliferation, apoptosis, EMT, invasion, and migration.

### miR-545 regulates the cell cycle

The cell cycle is closely related to cell proliferation and requires appropriate mitotic signals and an appropriate environment for normal cell proliferation.^50^ miR-545 can inhibit cell cycle progression by targeting four target genes *(CCND1, CCND2, CDK4,* and *CBLL1*), and it promotes cell cycle progression by targeting RIG-I. miR-545-3p induces G_0_/G_1_ phase arrest in cancer cell lines by targeting CCND1 and CDK4 in LC,^14^ CBLL1 in non-small cell lung cancer (NSCLC),^7^ and LAMC2 in OSCC.^39^ In CRC, miR-545-3p arrests the cell cycle at the G_1_ phase by targeting CDK4.^12^ miR-545-5p targets CCND2^44^ in NPC, causing G_0_/G_1_ phase arrest in the cell line.

### miR-545 regulates cell proliferation

The degree of cell proliferation is a key indicator of the pathways and mechanisms governing cell survival and death.^51^ Disruptions in the inhibitory pathways associated with proliferation can result in abnormal cell growth, a hallmark of malignancy.^50^ As shown in Supplementary Table 4, miR-545-3p inhibits cell proliferation by targeting 18 PCGs. In GBM/LGG and HCC cells, miR-545-3p promotes cancer cell proliferation by targeting LUZP1^4^ and SMAD7,^28^ respectively. miR-545-3p targets multiple genes and regulates tumor growth in xenograft animals. For example, it promotes tumor growth via four targets in LC (*CCND1*^14^ and *CDK4*^14^) and OC (*PLK1*^52^ and *KDM4B*^52^) but inhibits tumor growth in HCC via two targets (*RIG-I*^8^ and *MT1M*^53^). Additionally, miR-545-5p promotes tumor growth in animal models of NSCLC and SaOS by targeting c-Met^45^ and DIMT1,^9^ respectively.

The miR-545-3p/RIG-I axis inhibits the proliferation of OSCC (HSC4)^54^ and PDAC (HEK293, PANC1, and SW1990)^55^ cell lines while promoting the proliferation CRC DLD-1/HCT116^11^ cell lines. In CRC xenografts using LOVO cells in BALB/c nude mice, miR-545-3p promotes tumor growth^17^ but inhibits it in HT29/HCT116 cells.^18^ The impact of miR-545-3p on cell proliferation differs between various cancers owing to variations in gene regulatory networks. Further research is required to elucidate the underlying mechanisms.

### miR-545 regulates apoptosis

Apoptosis is a type of programmed cell death that can be initiated by intracellular or extracellular signals, which plays a crucial role in antitumor processes.^56^ miR-545-3p and miR-545-5p promote apoptosis by targeting ten and two genes, respectively. Additionally, miR-545-3p inhibits apoptosis by downregulating MT1M expression.

miR-545-3p promotes apoptosis by targeting 10 downstream PCGs, including CCND2 in NPC^57^; KU70^57^ and HDGF^6^ in LC; CBLL1,^7^ PRKCA,^15^ and YAP1 in NSCLC^26^; FAM98A in EC^32^; SLC7A11 in THCA^35^; PFKFB3 in CRC^29^; and PPA1 in OC.^32^ miR-545-5p promotes apoptosis by targeting SSFA2 in LC^36^ and MDM2 in CRC.^48^

Notably, miR-545-3p overexpression inhibits apoptosis by targeting MT1M in two ESCC cell lines (TE-1 and ECA109).^43^ The effects of miR-545-3p on apoptosis vary between different cancers, as they act as intermediate signal transducers and relay different upstream signals. Further research is required to fully understand these underlying mechanisms.

### miR-545 regulates epithelial-mesenchymal transition

EMT is a key process in tumor metastasis that enables tumor cells to invade blood or lymphatic vessels and generate circulating tumor cells.^58^^,^^59^ miR-545-3p inhibits EMT in NSCLC and CRC cells by targeting CBLL1^7^ and MYO6,^16^ which suppress the neural-like transformation of E-cadherin and vimentin expression. MiR-545-5p inhibits EMT in NPC cells by targeting CCND2, which prevents the reduction of E-cadherin and the replacement of the cytokeratin cytoskeleton with vimentin.^44^ Additionally, miR-545-5p downregulates biliverdin reductase by targeting c-Met, thereby inhibiting EMT in NSCLC cells.^45^

### miR-545 regulates cell invasion and migration

Invasion and migration are critical steps in tumor metastasis and major contributors to cancer mortality. Studies have shown that miR-545-3p inhibits cancer cell invasion and migration by targeting 21 genes, including *HK2, HDGF, PRKCA, HMGB3, YAP1, ZEB2, HS6ST2, EMS1, MYO6, VEGFA, PLK1, KDM4B, PPA1, CDK4, EIF3C, SLC7A11, WBP2, LAMC2, RIG-I,* and *GNB1.* Conversely, miR-545-3p promotes cancer cell invasion and migration by targeting three genes, LUZP1, SMAD7, and MT1M, and miR-545-5p promotes them by targeting six genes, including *CCND2, SSFA2, MET, CNPY2, MDM2,* and *DIMT1*. miR-545-3p inhibits cancer cell invasion and migration by targeting 20 genes including *HK2 in NB*^5^; *HDGF* in LC^6^; *PRKCA*,^15^ *HMGB*,^25^ *YAP1,*^26^ *ZEB2*,^10^ and *HS6ST2*^38^ in NSCLC; *EMS1*^40^ in GC; *MYO6*^16^ in CRC; *VEGFA* in EC^41^; *PLK1,*^52^ *KDM4B*,^52^ and *PPA1*^32^ in OC; *CDK4* in TNBC^13^; *EIF3C* in CxCa^34^; *SLC7A11* in THCA^35^ and *PDAC61*; *WBP2* in TNBC^33^; *LAMC2* and *RIG-I* in OSCC^39^^,^^54^; and *GNB1* in RB.^42^ miR-545-3p can inhibit tumor metastasis in nude mice by targeting *KDM4B a*nd *PLK1* in BALB/c nude mice transplanted with OC cells.^52^

In contrast, research has shown that miR-545-3p can promote cancer cell invasion and migration by targeting three genes: *LUZP1* in GBM/LGG,^4^ *SMAD7* in HCC,^28^ and *MT1M* in HCC.^53^ Additionally, miR-545-5p promotes cancer cell invasion and migration by targeting six genes: *CCND2* in NPC,^44^ *SSFA2* in LC,^36^ *c-MET* in NSCLC,^45^ *CNPY2* in GC,^61^ *MDM2* in CRC,^48^ and *DIMT1* in SaOS.^9^

### miR-545 regulates ferroptosis

Ferroptosis is a type of programmed cell death that differs from apoptosis. It occurs because of iron-dependent lipid peroxidation, and it is independent of caspase and RIPK1 activities. Ferroptosis plays a significant role in tumor suppression.^62^

The xc^−^ transport system is an amino acid antiporter that facilitates GSH synthesis and maintains redox homeostasis.^63^ *SLC7A11* is a membrane-localized light-chain subunit that forms an xc^−^ transport system with the heavy-chain subunit SLC3A2.^64^ *SLC7A11* overexpression can lead to tumorigenesis and ferroptosis resistance.^65^

In THCA, miR-545-3p targets *SLC7A11* to promote ferroptosis in FTC133 and TPC-1 cells. In CRC, miR-545-3p targets transferrin (TF) to render HT-29 or HCT-116 cell-based xenograft mouse models resistant to the ferroptosis inducer erastin. This inhibits abnormal ferroptosis signaling and promotes cancer progression.^18^

## miR-545 is involved in cancer signaling

**Figure 3:**
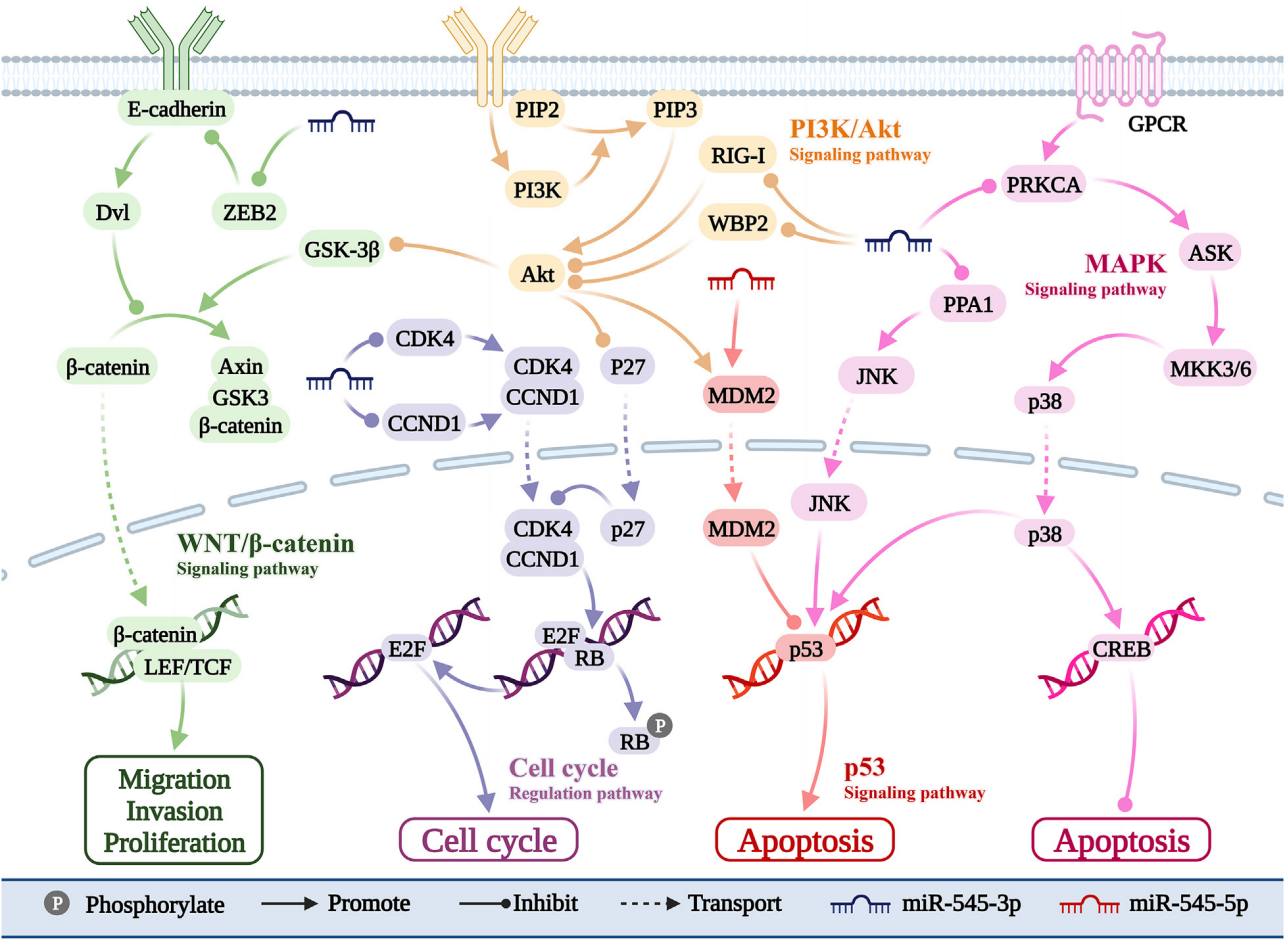
Unraveling the involvement of miR-545 in five signaling pathways and its impact on various biological functions. By targeting downstream genes, miR-545 regulates five pathways. These include the PI3K/AKT, MAPK, Wnt/β-catenin, cell cycle regulation, and p53 signaling pathways. Akt: Protein kinase B; ASK: Apoptosis signal-regulating kinase 1; CCND1: Cyclin D1; CDK4: Cyclin-dependent kinase 4; CREB: cAMP-response element binding protein; Dvl: Segment polarity protein dishevelled homolog DVL; E2F: Transcription factor E2F; GPCR: G protein coupled receptor; GSK-3: Glycogen synthase kinase 3; JNK: c-Jun N-terminal kinase; LEF/TCF: Lymphoid enhancer factor/T-cell factor; MAPK: Mitogen-activated protein kinase; miR: Micro ribonucleic acid; MKK3/6: Mitogen-activated protein kinase kinase 3/6; PI3K: Phosphoinositide-3-kinase; PIP2: Phosphatidylinositol 4,5-bisphosphate; PIP3: Phosphatidylinositol 3,4,5-triphosphate; PPA1: Inorganic pyrophosphatase 1; PRKCA: Protein kinase C alpha; RB: Retinoblastoma; RIG-I: Retinoic acid-inducible gene I; WBP2: WW domain Binding Protein 2; WNT: Wingless-related integration site; ZEB2: Zinc finger E-box binding homeobox 2.

As shown in Figure 3, miR-545-3p plays a role in several cancer-related signaling pathways. These include the protein wnt (Wnt)/β-catenin,^10^ PI3K/AKT^11^ cell cycle,^12^, ^13^, ^14^ and p38 pathways.^15^ Additionally, miR-545-5p participates in the p53 pathway by targeting MDM2.^48^

### Wnt/β-catenin signaling pathway

The Wnt/β-catenin pathway is crucial for stem cells.^66^ Abnormal activation of this pathway promotes tumor stem cell renewal, proliferation, and differentiation.^67^ ZEB2 is a transcription factor that functions alone or in combination with other proteins.^68^ In NSCLC, miR-545-3p inhibits ZEB2 expression and reduces free β-catenin protein levels in the cytoplasm. This prevents β-catenin from entering the nucleus and blocks the Wnt/β-catenin pathway. Consequently, cancer cell proliferation, invasion, and metastasis are inhibited.^10^^,^^69^

### PI3K/AKT signaling pathway

The PI3K/AKT pathway regulates cell phenotypes, such as proliferation, invasion, autophagy, and senescence. It is often abnormally activated in human cancers.^70^^,^^71^ RIG-I is an innate immune sensor that acts as a tumor suppressor.^72^ miR-545-3p targets and inhibits RIG-I expression in CRC and HCC. This activates the PI3K/AKT pathway and promotes cell proliferation.^11^ However, miR-545-3p inhibits cancer progression by targeting RIG-I in PDAC and OSCC.^54^^,^^55^ WBP2 is an oncoprotein that promotes AKT phosphorylation.^73^ miR-545-3p targets and inhibits WBP2 expression in TNBC. This inhibits PI3K/AKT pathway activation and slows cancer progression.^33^

### Mitogen-activated protein kinase signaling pathway

The mitogen-activated protein kinase (MAPK) pathway is important for cancer development and is frequently activated in various cancers.^74^ JNK^75^ and p38^76^ signals are key components of this pathway that affect cell proliferation, differentiation, and migration. PRKCA encodes PKCα, which promotes p38 phosphorylation and activates the p38 pathway via MKK3/6.^77^^,^^78^ In NSCLC, miR-545-3p inhibits p38 pathway activation by targeting PRKCA.^15^ It also inhibits cancer cell invasion and promotes apoptosis. PPA1 encodes an inorganic pyrophosphatase dysregulated in several cancers.^79^ PPA1 activates the JNK pathway and promotes NSCLC progression in a TP53-dependent manner.^80^ In OC, miR-545-3p suppresses tumor malignancy by targeting PPA1 to inhibit JNK pathway activation.^32^

### Cellular tumor antigen p53 signaling pathway

*TP53* is a crucial tumor suppressor gene that encodes p53. This protein is deleted or mutated in more than half of all cancers.^81^^,^^82^ p53 regulates cell cycle arrest and apoptosis.^83^^,^^84^ MDM2 is an E3 ubiquitin ligase and a major antagonist of p53.^85^ It cooperates with MDMX to ubiquitinate p53, leading to its degradation and nuclear export. This inhibits p53 transcriptional activity.^86^ In CRC, miR-545-5p inhibits MDM2 expression, increases p53 transcriptional activity, and inhibits tumor growth.^48^

### Cell cycle regulation pathway

The cell cycle in cancer metabolism is complex, and its dysregulation is a key feature of cancer progression.^87^ The cell cycle pathway regulates cell proliferation and affects cancer progression. This is an important target in cancer therapy.^88^ Various cyclin/CDK complexes play crucial roles in regulating the cell cycle pathway.^89^^,^^90^ The CCND1/CDK4 complex phosphorylates RB1 and promotes E2F transcriptional activity. This promotes the G1/S transition of cells.^91^^,^^92^ The cell cycle is regulated by upstream pathways, such as PI3K/AKT^93^ and p53 signaling.^94^ In CRC and TNBC, miR-545-3p targets CDK4 to inhibit E2F transcriptional activity and causes G1 arrest in cancer cells.^12^^,^^13^ In LC, miR-545-3p inhibits cancer cell proliferation by targeting CCND1 and CDK4.^14^

## miR-545 has prognostic value in cancer

As shown in Table 1, abnormal expression of miR-545 is substantially associated with the prognosis and clinicopathological features of cancer patients. Thus, it can serve as a biomarker for cancer prognosis. In NSCLC, PDAC, and OC, the low miR-545 expression indicates poor OS, progression-free survival (PFS), and DFS.

**Table 1:** Prognostic value of miR-545-3p.

Origin of mature miR-545	Cancer	Sample size, *n*	miR-545 expression	Clinicopathological characteristics	Prognostic value	Reference
3p	NSCLC	84	Down-regulation	Associated with lymph node metastasis↑, and tumor stage↑	Shorter OS	10
	PDAC	78	Down-regulation	–	Shorter OS and DFS	55
	OC	60	Down-regulation	Associated with pTNM stage↑	Shorter OS	52
		218	Down-regulation	Associated with response status↓	Shorter OS and PFS	95

DFS: Disease-free survival; HCC: Hepatocellular carcinoma; NSCLC: Non-small-cell lung cancer; OC: Ovarian cancer; OS: Overall survival; PDAC: Pancreatic ductal adenocarcinoma; PFS: Progression-free survival; pTNM: Pathological tumor node metastasis; TNM: Tumor node metastasis.

In NSCLC, low miR-545-3p expression is associated with more severe lymph node metastasis and a higher tumor stage. This predicts a shorter OS.^10^ In PDAC, patients with low miR-545-3p expression have poor OS and DFS.^55^ In OC, low miR-545-3p expression is associated with poor OS and PFS. It is also strongly associated with a higher pTNM stage and stronger platinum drug resistance.^52^^,^^95^

Current research on the clinical value of miR-545 is limited, and it has mostly focused on miR-545-3p. Further studies with larger sample sizes are required to understand the association between miR-545 expression and the prognosis of cancer patients. The potential differences between miR-545-3p and miR-545-5p expression in association with patient prognosis need to be determined.

## miR-545 and cancer therapy

miRNAs can influence cancer therapy by participating in extensive regulatory networks.^96^ miR-545 mediates the effects of chemical factors, such as formononetin,^13^ and physical factors, such as ionizing radiation,^97^ on cancer cells. It can target downstream factors, such as YAP1, HK2, PFKFB3, KU70, and C–C motif chemokine ligand 22 (CCL22), to inhibit cell resistance to chemotherapy and radiotherapy. MiR-545 can also promote resistance to chemotherapy and radiotherapy via MT1M. Additionally, miR-545 enhances immune cell proliferation and infiltration by inhibiting downstream factors, such as TIM-3 and CCL22.

### miR-545 and chemotherapy

**Figure 4:**
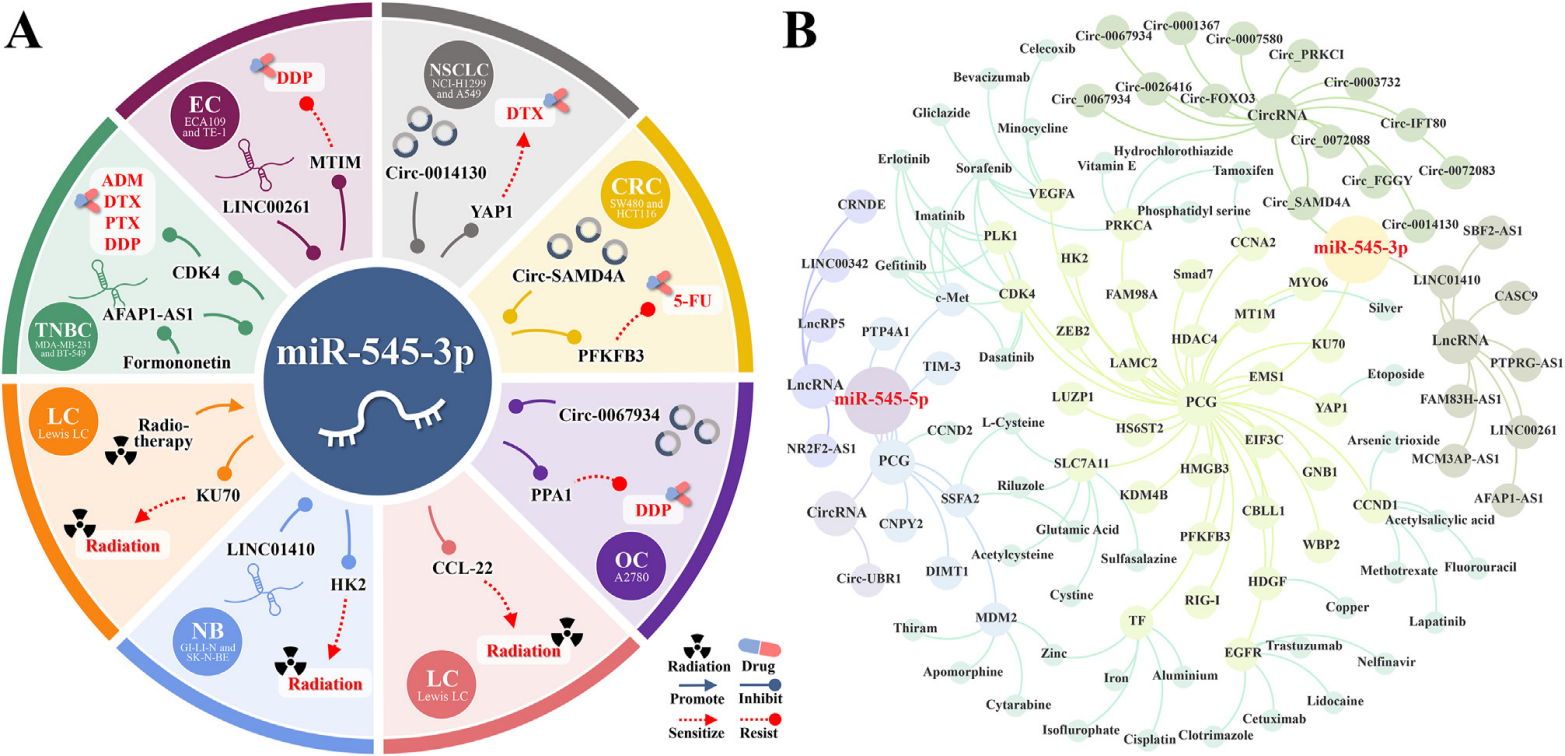
miR-545 has a significant impact on cancer cell sensitivity to radiation and chemotherapy. (A) miR-545 is linked to cancer cell resistance to radiotherapy and five chemotherapeutic drugs (DDP, ADM, DTX, PTX, and 5-Fu). (B) The ceRNA/miR-545/PCG axes and therapeutic drugs targeting miR-545 downstream targets can be found in the CADDIE database. 5-FU: 5-fluorouracil; ADM: Adriamycin; AFAP1: Actin filament associated protein 1; AS1: Antisense RNA 1; CBLL1: Cbl proto-oncogene like 1; CCNA2: Cyclin A2; CCND1: Cyclin D1; CCND2: Cyclin D2; CDK4: Cyclin-dependent kinase 4; CircRNA: Circular RNA; c-Met: Mesenchymal-epithelial transition factor; CNPY: Canopy FGF signaling regulator; CRC: Colorectal cancer; CRNDE: Colorectal neoplasia differentially expressed; DDP: Cisplatin; DTX: Docetaxel; EC: Endometrial cancer; EGFR: Epidermal growth factor receptor; EMSI: ; EMT: Epithelial-mesenchymal transition; ESCC: Esophageal squamous cell carcinoma; FAM98A: Family with sequence similarity 98 member A; FGGY: GGY carbohydrate kinase domain containing; FOXO3: Transcription factor forkhead box O-3; GC: Gastric cancer; GNB1: G protein subunit beta 1; HCC: Hepatocellular carcinoma; HDGF: Heparin binding growth factor; HK2: Hexokinase II; HMGB3: High-mobility group box 3; IFT: Intraflagellar transport; HK2: Hexokinase 2; KU70: X-ray repair cross complementing 6; LAMC: Laminin subunit gamma 2; LC: Lung cancer; LINC: Long intergenic noncoding; LncRNA: Long non-coding ribonucleic acid; LUZP1: Leucine zipper protein 1; MCM3AP: Minichromosome maintenance complex component 3 associated protein; MDM2: Murine double minute 2; miR: Micro ribonucleic acid; NB: Neuroblastoma; NPC: Nasopharyngeal carcinoma; NRF2F2: Nuclear factor erythroid 2-related factor 2; NSCLC: Non-small cell lung cancer; OC: Ovarian cancer; OSCC: Oral squamous cell carcinoma; PCG: protein-coding gene; PDAC: Pancreatic ductal adenocarcinoma; PFKFB3: 6-phosphofructo-2-kinase/fructose-2,6-biphosphatase 3; PLK1: Polo like kinase 1; PPA1: Inorganic pyrophosphatase 1; PRKCA: Protein kinase c alpha; PTX: Paclitaxel; RB: Retinoblastoma; RIG-I: Retinoic acid-inducible gene I; SAMD4A: Sterile alpha motif domain containing 4A; Smad: Small mother against decapentaplegic; TC: Thyroid carcinoma; TIM-3: T-cell immunoglobulin and mucin-domain containing-3; TNBC: Triple-negative breast cancer; UBR1: Ubiquitin protein ligase E3 component N-Recognin 1; VEGFA: Vascular endothelial growth factor A; WBP2: WW domain binding protein 2; YAP1: Yes1 associated transcriptional regulator; ZEB2: Zinc finger E-box binding homeobox 2; ZFR: Zinc finger RNA binding protein.

Chemotherapy is a supplementary treatment for advanced cancers that prevents tumor cell growth.^98^ Figure 4A shows that formononetin indirectly increases miR-545 levels and improves cell sensitivity to four chemotherapeutic drugs (adriamycin, docetaxel DTX, paclitaxel, and cisplatin DDP). By targeting YAP1, miR-545 increases cell sensitivity to DTX. Targeting HT1M and PPA1 increases cell resistance and sensitivity to DDP. Targeting PFKFB3 increases cell sensitivity to 5-fluorouracil (5-FU).

Formononetin is a 7-hydroxyisoflavone with a methoxy group that can be found in red clovers and the Chinese herb *Astragalus membraneceus*.^99^ It can regulate cell death and cell cycle processes, and it shows promise in preventing and treating cancer.^100^ Formononetin inhibits the growth, invasion, and movement of two TNBC cell lines (MDA-MB-231 and BT-549) by decreasing AFAP1-AS1 expression, increasing miR-545-3p levels, and improving sensitivity to the four drugs mentioned above.^13^

DTX is a chemotherapeutic drug in the taxane family commonly used to treat NSCLC patients.^101^ In NSCLC, DTX-resistant NCI–H1299 and A549 cell lines exhibit lower miR-545-3p expression levels than non-resistant NCI–H1299 and A549 cell lines.^26^ High miR-545-3p expression in the circ _0014130/miR-545-3p/YAP1 axis reduces DTX resistance in two NSCLC cell lines (NCI–H1299 and A549).^26^

DDP is a stable platinum coordination compound at normal temperature and pressure that can be used as a standard treatment for ESCC.^102^ In ESCC, miR-545-3p is a part of the LINC00261/miR-545-3p/MT1M axis that increases DDP resistance in two ESCC cell lines (TE-1 and ESCC109).^43^ In OC, miR-545-3p involvement in the Circ_0067934/miR-545/PPA1 pathway reduces DDP resistance in the A2780/DDP cell line.^32^

In addition, 5-FU, a naturally occurring uracil analog,^103^ is often used as a nanoagent to treat different types of cancers, including CRC and breast cancer.^104^ Highly expressed miR-545-3p participated in the Circ_SAMD4A/miR-545/PFKFB3 axis in two CRC cell lines (SW480/5-FU and HCT-116/5-FU) and inhibited 5-FU resistance.^29^

### miR-545 and radiotherapy

Radiotherapy uses ionizing radiation to treat cancer by targeting cells and molecules.^105^ It increases miR-545 expression, which inhibits KU70, HK2, and CCL22 expression and enhances tumor radiosensitivity Figure 4A. In Lewis lung carcinoma cells, radiotherapy increases miR-545-3p expression in irradiated areas and inhibits tumor progression by reducing CCL22 expression.^106^ High miR-545-3p levels also enhance radiosensitivity in C57BL/6 mice by reducing KU70 expression.^57^ In the NB cell lines GI-LI-N and SK-N-BE(2), high levels of miR-545-3p increase radioresistance by inhibiting glycolysis via the LINC01410/miR-545-3p/HK2 axis.^5^

### miR-545 and immunotherapy

**Figure 5:**
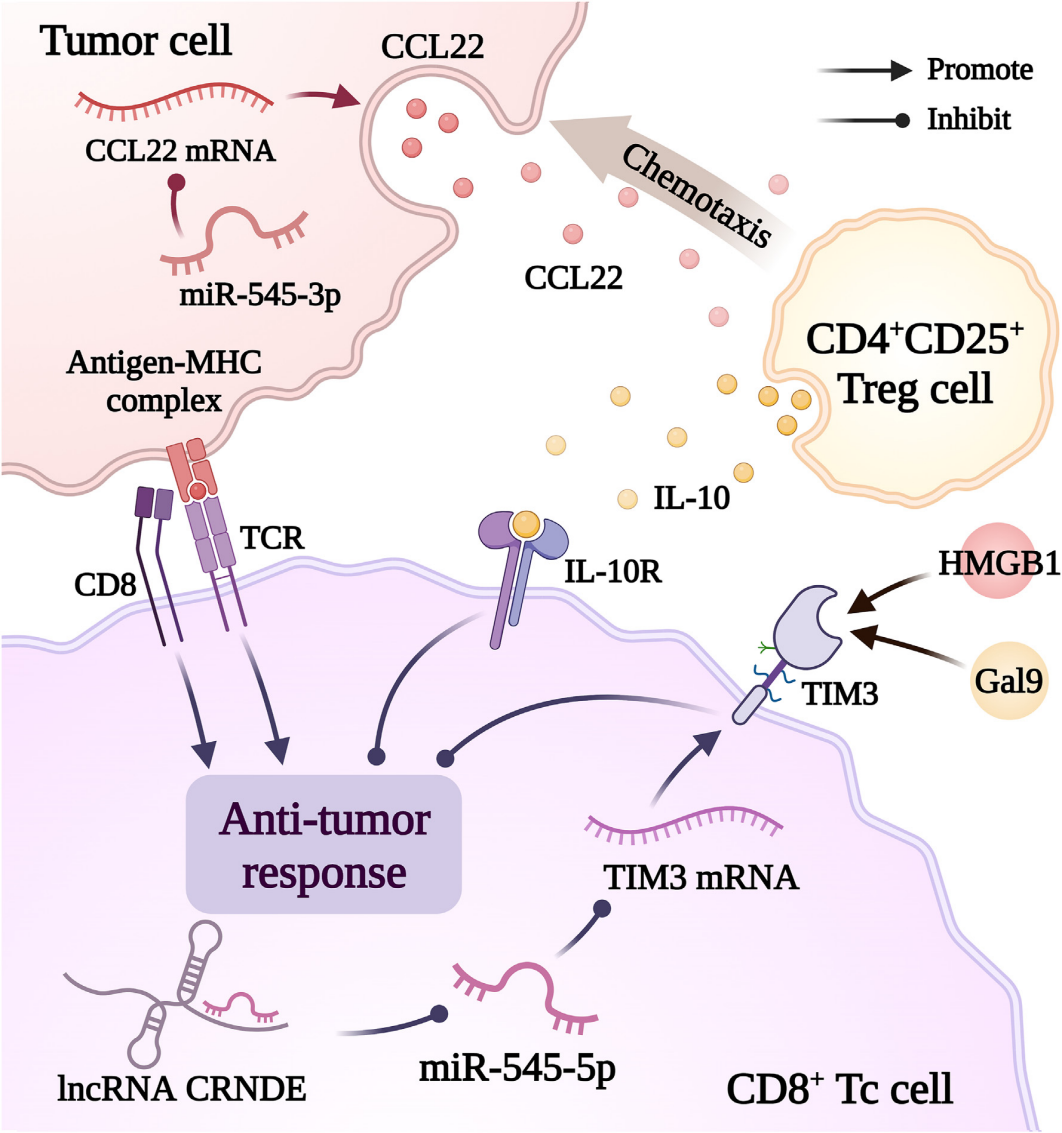
Investigating the role of miR-545 in shaping the tumor immune microenvironment. miR-545 can influence the interaction between tumor cells and immune cells (CD4^+^CD25^+^ regulatory T cells and CD8^+^ cells) by reducing CCL22 and TIM-3 levels. CCL22: CC Chemokine ligand 22; CD: Cluster of differentiation; CRNDE: Colorectal neoplasia differentially expressed; Gal9: Galectin-9; HMGB1: High mobility group box 1; IL: Interleukin; lncRNA: Long non-coding ribonucleic acid; MHC: Major histocompatibility complex; miR: Micro ribonucleic acid; mRNA: Messenger ribonucleic acid; TCR: T-cell receptor; TIM-3: T-cell immunoglobulin and mucin-domain containing-3; Treg cell: Regulatory T cell.

Cancer cells can evade or suppress the immune system by altering their antigens^107^ and modifying the tumor microenvironment.^108^^,^^109^ Immunotherapy is a novel cancer treatment that induces antitumor immune responses.^110^ As shown in Figure 5, miR-545 enhances antitumor immunity by reducing TIM-3 and CCL22 levels.

TIM-3 is an immune checkpoint that negatively regulates immunity in various cancers (CRC, CxCa, and GC) and leukemia stem cells.^111^ Co-expression of PD-1 reduces the effectiveness of tumor immunotherapy and promotes tumor growth.^112^^,^^113^ In OSCC, miR-545-5p reduces TIM-3 expression to enhance immunity and inhibit tumor growth.^47^

A large number of regulatory T cells can suppress immunity and worsen cancer prognosis.^114^ CCL22 increases regulatory T cell (Treg) infiltration in tumors and promotes tumor growth.^115^ In Lewis lung carcinoma, miR-545-3p recruits CD4^+^CD25^+^ regulatory T cells via CCL22 to inhibit tumor growth.^106^

### miR-545–related therapeutic drugs

As shown in Figure 4B, a search of the CADDIE database (https://exbio.wzw.tum.de/caddie/)^116^ revealed that several approved drugs target miR-545 downstream of PCGs. These include apomorphine, cytarabine, zinc, and thiram targeting MDM2, as well as bevacizumab, sorafenib, celecoxib, minocycline, and gliclazide targeting VEGFA. Further research is needed to understand the interactions between miR-545 and these PCG-targeted drugs to develop new combination therapies for clinical use.

## Discussion

MiR-545, located on chromosome Xq13.2, has two mature forms: miR-545-3p and miR-545-5p. Extensive evidence has demonstrated the dysregulation of miR-545 expression in various cancers, making it a promising cancer biomarker. The distinct expression patterns of miR-545 in different tumor types may be attributed to its participation in diverse regulatory networks and varying roles in different cancer contexts. However, comprehensive studies outlining the regulatory landscape of miR-545 in different cancers are lacking. Thus, it is imperative to map the regulatory mechanisms of miR-545 in diverse tumor types in future studies. Although dysregulated miR-545 expression in most tumors has been extensively explored, its differential expression in CRC remains controversial. This discrepancy may stem from factors such as cellular and tissue sample heterogeneity or differences in detection methods. Furthermore, findings from pan-cancer analyses using large databases, such as TCGA, do not align with experimental data consistently. This discrepancy could be attributed to factors such as the data volume within the database, the differential analysis algorithms employed, or the inherent heterogeneity of the experimental samples. To address these challenges, future research endeavors could increase the sample size and diversity and reduce error rates by integrating data from additional databases, such as the Gene Expression Omnibus (GEO), for joint analysis. Moreover, the expression level of miR-545 could be precisely measured using advanced chips or sequencing technologies. Concurrently, expanding the repertoire of verified cell lines and increasing their numbers would contribute to obtaining more robust and convincing results. Notably, there are discrepancies in the expression patterns of miR-545-3p and miR-545-5p, possibly stemming from insufficient data on certain tumor types in existing studies. Hence, future investigations should focus on elucidating the differential expression of miR-545 in diverse tumor subtypes, stages, clinical features, and prognoses. Leveraging multi-source data and employing multilevel analysis methods would enable a comprehensive assessment of the potential diagnostic value of miR-545 in various tumors.

MiR-545 is located within intron 1 of the lncRNA *FTX*. Analysis of the TCGA database revealed a significant correlation between miR-545 and *FTX*, as well as other miRNA clusters in certain tumors. However, this correlation was not significant in most TCGA tumors, and *FTX* CpG island methylation levels did not substantially influence miR-545 expression. This discrepancy may be attributed to missing data in the database. To elucidate the role and mechanisms of the lncRNA *FTX* and miR-545, along with their adjacent genes, in tumorigenesis, future research could enhance data availability by integrating databases or incorporating self-owned samples. Additionally, comprehensive analyses integrating genomics, transcriptomics, proteomics, metabolomics, and other data can be employed to investigate the multifaceted functions of miR-545 at various levels. Network analysis, systems biology, machine learning, and other methodologies can be applied to construct regulatory networks and functional modules associated with the lncRNA *FTX* and miR-545. Subsequently, a series of molecular biology experiments should be conducted to further investigate the mechanisms of their interaction. In recent years, sex disparities in tumorigenesis and disease progression have attracted increasing attention. Studies have indicated that fat accumulation or distribution exhibits varying effects on colorectal, esophageal, and liver cancers depending on sex.^117^ Therefore, it is crucial to consider the unique chromosomal locations of *FTX* and miR-545, as well as their potential sex-specific implications in tumor diagnosis and treatment. Nevertheless, TCGA results only revealed sex differences in miR-545 expression in a few tumor types, and the current study did not focus on cell line selection based on sex. Future investigations should collect gene expression profiles from patient tissues of different sexes and incorporate clinical data from male and female cancer patients for comprehensive statistical analysis. Furthermore, the expression levels and biological functions of miR-545 were validated using *in vitro*-cultured cancer cell lines and animal models encompassing both males and females.

miR-545 is competitively inhibited by 15 circRNAs and 12 lncRNAs, actively participating in five tumor-associated pathways that regulate key tumor cell behaviors affecting tumor progression, including cell cycle, proliferation, apoptosis, EMT, invasion, and migration. Although the initial discovery of the miR-545 regulatory network in tumors is promising, most mechanisms identified thus far involve single ceRNA axes, highlighting the need for more systematic and comprehensive network-based studies. Additionally, given the intricate mechanism of action of miRNAs, research on miR-545 should not solely focus on the inhibition of protein translation at the post-transcriptional level but also explore the mechanisms underlying the induction of target RNA degradation. To address these challenges, future studies should integrate bioinformatic analysis with *in vivo* and *in vitro* experiments to explore the regulatory network involving miR-545 and its associated molecules more comprehensively. This will help to elucidate their potential as therapeutic targets, thereby establishing a foundation for the development of more precise and targeted therapeutic strategies.

The extensive differential expression patterns and regulatory mechanisms of miR-545 highlight its potential clinical applications. This review revealed a significant correlation between abnormal miR-545 expression and the prognostic and clinicopathological features of various tumors. However, considering the ambiguity surrounding miR-545 expression, its reliability as a stable biomarker warrants further investigation. Future studies should collect extensive clinical data, conduct cohort analyses, and perform validation studies to assess the potential of miR-545 as a reliable biomarker, either alone or in combination with other genes. Moreover, although miR-545 has been implicated in interactions with multiple tumor treatments, such as chemotherapy, radiotherapy, and immunotherapy, via at least 10 downstream factors, research on its interactions with common treatments remains limited. For instance, the role of miR-545 in cancer immunity, including its relationship with immune checkpoints and immune cell functions, requires further exploration. Future studies should expand their scope to include a variety of treatments and drugs and monitor changes in miR-545 expression pre- and post-treatment to gain a better understanding of its involvement in chemotherapy and radiotherapy resistance. RNA therapy offers advantages, such as high therapeutic efficacy, low drug toxicity, strong specificity, and broad applicability.^118^ Given the involvement of miRNAs in diverse regulatory networks and their close association with normal physiological processes and cancer development, therapeutic strategies targeting miRNAs hold significant promise, and several miRNA-based therapeutic approaches already exist. For instance, rMMN nanomedicines loaded with miR-30a-5p upregulated miR-30a-5p levels both *in vitro and in vivo*, thereby suppressing the malignant phenotype of ocular melanoma by targeting the transcription factor E2F7.^119^ Numerous therapeutic approaches targeting miRNAs and miRNA mimics have been used in clinical research.^120^ Future research should elucidate the regulatory modes of miR-545, develop appropriate drug delivery vehicles and methods, and ultimately achieve the clinical translation of miR-545 with high efficiency and low side effects.

MiR-545 exhibits differential expression patterns across various tumors and plays a pivotal role in intricate regulatory networks that influence tumor initiation and progression. In the future, we anticipate increased research emphasis on miR-545 and its target interactions, aiming to unravel its molecular mechanisms, therapeutic potential, and diagnostic value. Such investigations will provide valuable insights into the clinical applications of miR-545 in cancer and establish a robust theoretical foundation for subsequent advancements in clinical diagnosis and treatment. Given its potential as a promising diagnostic and therapeutic molecule, miR-545 warrants further comprehensive investigation and in-depth studies.

## Conclusions

This review systematically examines miR-545 and its dysregulated expression in cancer cells. It summarizes the regulatory role and molecular mechanisms of miR-545 in cancer and its relationship with patient prognosis and drug treatments. This review highlights the potential of miR-545 as a biomarker and therapeutic target for human cancers and identifies gaps in the current research to guide future studies. MiR-545 is an important regulatory molecule with potential practical applications in clinical cancer treatment.

## Funding

This study was supported by the Qiantang Scholars Fund of the Hangzhou City University (No. 210000-581835).

## Authors contribution

Jinze Shen: Conceptualization, Writing - Original Draft, Visualization; Xinming Su: Writing - Original Draft; Qurui Wang: Visualization; Yufei Ke: Visualization; Tianyu Zheng: Visualization; Yunan Mao: Visualization; Zehua Wang: Visualization; Jingyin Dong: Writing - Review & Editing; Shiwei Duan: Conceptualization, Writing - Review & Editing, Funding acquisition.

## Ethics statement

None.

## Data availability statement

All data generated or analyzed during this study are included in this published article and its supplementary information files.

## Conflict of interest

The authors declare that they have no known competing financial interests or personal relationships that could have appeared to influence the work reported in this paper.
