# Supplementary material for: Current and future perspectives on the regulation and functions of miR-545 in cancer development
Source: Cancer Pathog Ther. 2023 Sep 5;2(3):142–54. doi: 10.1016/j.cpt.2023.09.001 (PMC11252520; doi:10.1016/j.cpt.2023.09.001)
Supplement: Multimedia component 1 [file mmc1.docx]

**Supplementary Materials**

**TEXT**

Pan-cancer analysis of (micro ribonucleic acid) miR-545

miR-545 maturation: From stem-loop to mature strand

Potential causes of inconsistencies between The Cancer Genome Atlas (TCGA) data analysis and existing research on miR-545

miR-545 and its host gene *FTX*

Correlation between miR-545 and other miRNAs encoded in *FTX*

**Supplementary** **Table 1.** Dysregulated miR-545 expression in human cancers

**Supplementary** **Table 2.** Comparison of TCGA data analysis and existing research on miR-545

**Supplementary** **Table 3.** miR-545–related competitive endogenous ribonucleic acid (ceRNA) axes in cancer

**Supplementary** **Table 4.** Effects of miR-545 *in vivo* and *in vitro*

**Supplementary** **Figure 1.** Pan-cancer analysis of miR-545.

**Supplementary** **Figure 2.** miR-545 expression differences between males and females.

**Supplementary** **Figure 3.** Chromosomal location of pre-miR-545 and its host gene *FTX*.

**Supplementary** **Figure 4.** Correlation between pre-miR-545 expression levels and *FTX* in different sexes.

**Supplementary** **Figure 5.** Correlation between pre-miR-545 and other miRNA clusters in *FTX*.

**References**

**TEXT**

**Pan-Cancer Analysis of miR-545**

We retrieved expression data (RPM) for the mature chain of miR-545, including both miR-545-5p and miR-545-3p, from the pan-cancer dataset in The Cancer Genome Atlas (TCGA) using the University of California Santa Cruz (UCSC) Xena database (https://xenabrowser.net).^[^^1,2]^ After removing samples with zero expression, we transformed the expression levels using log2(RPM+1) and analyzed the miR-545 expression data across 31 different tumors.

We determined the median percentile of miR-545 expression among all non-zero-expressed miRNAs in pan-cancer samples. Supplementary Figure 1A shows high miR-545-5p expression in 28 tumors (0.5–0.75 quantile, Q3) and intermediate expression in three tumors (0.25–0.5 quantile, Q2) out of the 31 tumors analyzed. In contrast, miR-545-3p had high expression in two tumors (0.5–0.75 quantile, Q3), intermediate expression in 23 tumors (0.25–0.5 quantile, Q2), and low expression in six tumors (0–0.25 quantile, Q1).

Supplementary Figure 1B shows data comparing tumor samples to paracancerous samples for 20 different cancer types. We used an unpaired Wilcoxon test in R software version 4.1.3 to analyze the differences in miR-545 expression between these samples. After adjusting for multiple comparisons using the Bonferroni method for 20 different cancer types, miR-545-5p expression was significantly upregulated in four tumor types (head and neck squamous cell carcinoma [HNSC], lung adenocarcinoma [LUAD], lung squamous cell carcinoma [LUSC], and uterine corpus endometrial carcinoma [UCEC]), with a Bonferroni-adjusted *P* value < 0.05. Additionally, miR-545-3p expression was significantly upregulated in one type of tumor (UCEC).

Given that miR-545 is located on the X chromosome, we considered the possibility that sex differences may influence its expression. Previous studies have indicated that miR-545-3p expression is higher in male hepatocellular carcinoma (HCC) patients than in female HCC patients.^[3]^ To further investigate this, we obtained patient sex information from TCGA pan-cancer dataset using the UCSC Xena database and divided all cancer samples into male and female groups. We then used an unpaired Wilcoxon test to compare miR-545 expression between samples from different sexes across various cancer types. Our results indicate that sex exerted no significant effect on miR-545 expression in most tumors [Supplementary Figure 2].

**miR-545 Maturation: From Stem-Loop to Mature Strand**

miR-545-5p and miR-545-3p originate from the 5′ and 3′ ends of the pre-mir-545 stem-loop structure, respectively, according to miRbase (https://www.mirbase.org/).^[4]^ We retrieved the expression data for the pre-mir-545 stem-loop structure from TCGA pan-cancer dataset using the UCSC Xena database and transformed the data using log2(RPM+1). We calculated the correlation between pre-mir-545 and both miR-545-5p and miR-545-3p using Pearson’s correlation. Supplementary Figure 1 shows that in TCGA dataset, miR-545-5p expression was higher than that of miR-545-3p. Additionally, the correlation between pre-mir-545 and miR-545-5p was stronger than that between pre-mir-545 and miR-545-3p [Supplementary Figure 3]. Based on these results, we believe that in TCGA database, most pre-miR-545 tends to mature into miR-545-5p, whereas only a small portion matures into miR-545-3p. Supplementary Figure 3 also reveals that in adrenocortical carcinoma as well as pheochromocytoma and paraganglioma, pre-miR-545 was significantly positively correlated with miR-545-5p but not with miR-545-3p, suggesting that more pre-miR-545 matures into miR-545-5p in these cancer types. However, in CHOL (*n* = 4), kidney chromophobe (KICH; *n* = 4), and ovarian serous cystadenocarcinoma (OV; *n* = 3), there was no significant correlation between pre-miR-545 and either miR-545-5p or miR-545-3p, which may be attributed to the small sample size used.

**Potential Causes of Inconsistencies Between TCGA Data Analysis and Existing Research on miR-545**

Supplementary Table 1 shows that in TCGA database, miR-545-5p expression was significantly upregulated in four types of tumors (HNSC, LUAD, LUSC, and UCEC) with a Bonferroni-adjusted *P* value < 0.05. However, previous research has reported that miR-545-5p expression is downregulated in nasopharyngeal carcinoma (NPC) and non-small-cell lung cancer and that miR-545-3p expression is downregulated in EC, which is inconsistent with TCGA results. Several factors may contribute to these discrepancies, including differences in gene expression detection methods (real-time quantitative polymerase chain reaction (RT-qPCR) in previous studies *vs.* RNA-seq in TCGA database), a smaller number of control samples in TCGA database, heterogeneity of gene expression in tissue samples, or variations in cancer molecular subtypes.

In TCGA database, the proportion of samples with loss of miR-545-3p expression (72.0%) was much higher than that of miR-545-5p (33.7%). This discrepancy may be attributed to the low sequencing depth or coverage in earlier TCGA samples or technical limitations that resulted in a lack of sequencing sites. These factors may contribute to the inconsistency between TCGA data and previous research on miR-545.

TCGA results suggested that most pre-miR-545 matures into miR-545-5p. However, this was not consistent with the previous sequencing results from miRBase, which indicated that the average abundance of miR-545-3p was higher than that of miR-545-5p. This discrepancy may also be attributed to uneven library construction in TCGA database, resulting in low miR-545-3p abundance.

**miR-545 and its Host Gene *FTX***

We retrieved the expression data for *FTX* and XIST from TCGA pan-cancer dataset using the UCSC Xena database and transformed the data using log2(TPM+0.001). We then calculated the correlation between *FTX* and XIST with miR-545-5p, miR-545-3p, and pre-mir-545 using Pearson correlation.

In most cancers, miR-545 expression levels were not significantly different, and there was no significant correlation with *FTX* or XIST [Supplementary Figures 1B and 3]. However, miR-545-5p was highly expressed in HNSC, LUAD, LUSC, and UCEC, and it was significantly negatively correlated with *FTX* expression levels in COAD, KICH, LUAD, and prostate adenocarcinoma. In KICH, it was also significantly negatively correlated with XIST expression. MiR-545-3p was significantly highly expressed only in UCEC, and it was significantly negatively correlated with *FTX* expression levels in HNSC, mesothelioma (MESO), rectum adenocarcinoma (READ), and thymoma (THYM). It was also significantly negatively correlated with XIST expression THYM (*P* < 0.05, absolute value of *r* > 0.3).

CpG islands (CGIs) are short DNA sequences with a higher frequency of CG sequences than other regions of the genome.^[5]^ They are primarily found in the promoter and first exon regions of genes. Abnormal *CGI* methylation has been linked to tumorigenesis. Using TCGA database, we conducted a correlation analysis between the expression levels of miR-545 and *FTX*, the expression level of the downstream gene *XIST*, and the methylation levels of *FTX* CGIs [Supplementary Figures 3 and 4].

For most cancers, there was no significant relationship between the expression levels of pre-mir-545 and methylation of the *FTX* gene *CGI*. However, in males, cg04667267 in breast invasive carcinoma (BRCA), cg27556136 in kidney renal clear cell carcinoma (KIRC), and cg08195522 in skin cutaneous melanoma (SKCM) were significantly positively correlated with *FTX* (*P* < 0.05, absolute value of *r* > 0.3). In contrast, cg22690030 in KICH, cg17674548 in KIRC, cg15376401 in MESO, cg04624564 in KIRC, and cg07780887 in KICH and KIRC were negatively correlated with *FTX* levels (*P* < 0.05, absolute value of *r* > 0.3).

Additionally, in females, cg04667267, cg08195522, cg22690030/cg01076406/cg01829822, and cg27556136 in OV, acute myeloid leukemia (LAML), THYM, and uveal melanoma (UVM), respectively, were significantly positively correlated with *FTX* (*P* < 0.05, absolute value of *r* > 0.3). In contrast, cg26533737 in MESO and UVM, cg22690030 in esophageal carcinoma (ESCA), cg17674548 in KICH, cg01076406 in lymphoid neoplasm diffuse large B-cell lymphoma and ESCA, cg27556136/cg07780887 in SKCM, and cg04624564 in SKCM and THYM were significantly negatively correlated with *FTX* levels (*P* < 0.05, absolute value of *r* > 0.3).

**Correlation Between miR-545 and Other miRNAs Encoded in *FTX***

There are two miRNA clusters within the *FTX* intron: the miR-374b/421 cluster, which includes miR-421, miR-374b, and miR-374c, and the miR-545/374a cluster, which includes miR-545 and miR-374a. The expression data for pre-miR-545, pre-miR-421, pre-miR-374b, pre-miR-374c, and pre-miR-421 from TCGA pan-cancer dataset via the UCSC Xena database were transformed using log2(RPM+1). In various cancers, pre-miR-545 expression levels were significantly positively correlated with other miRNA clusters in *FTX* [Supplementary Figure 5]. These included pre-miR-374a in bladder urothelial carcinoma (BLCA), KIRC, kidney renal papillary cell carcinoma (KIRP), OV, pancreatic adenocarcinoma (PAAD), stomach adenocarcinoma (STAD), and testicular germ cell tumors (TGCT); pre-miR-374b in BLCA, BRCA, KICH, KIRP, LAML, brain lower-grade glioma (LGG), liver hepatocellular carcinoma, LUAD, LUSC, OV, STAD, TGCT, and UCEC; pre-miR-374c in COAD, HNSC, KIRP, LGG, LUAD, LUSC, PAAD, READ, and UCEC; and pre-miR-421 in BLCA, BRCA, KIRP, LUSC, OV, STAD, TGCT, THYM, and UCEC (*P* < 0.05 and *r* > 0.3).

**Supplementary** **Table 1: Dysregulated miR-545 expression in human cancers.**

| **Origin of mature miR-545** | **Cancer** | **Expression** | **Tumor cell *vs.* normal cell** | **Tissue or serum** | **Ref.** |
| --- | --- | --- | --- | --- | --- |
| 3p | BCa | Downregulated | — | Tumor tissues from 60 BCa patients *vs.* paracancerous tissues | ^[6]^ |
|  | CRC | Downregulated | Male cancer cell lines (HCT-8, HCT-116, DLD-1, and SW480) *vs.* male normal cell lines (NCM460) | Tumor tissues from 43 CRC patients *vs.* paracancerous tissues | ^[7]^ |
|  |  | Downregulated | — | Tumor tissues from 100 CRC patients *vs.* paracancerous tissues | ^[8]^ |
|  |  | Upregulated | — | Serum samples from pre-surgery patients with CRC (GSE40247) *vs.* serum samples from healthy people | ^[9]^ |
|  |  | Upregulated | Male cancer cell lines (HCT-116 and LoVo) and female cancer cell lines (HT-29) *vs.* male normal cell lines (NCM460) | — | ^[10]^ |
|  | EC | Downregulated | Female cancer cell lines (HEC-1B, EEC, and Ishikawa) *vs.* female normal cell lines (hESC) | Tumor tissues from 35 EC patients *vs.* paracancerous tissues | ^[11]^ |
|  |  | Downregulated | — | Tumor tissues from 73 EC patients *vs.* paracancerous tissues | ^[12]^ |
|  | GBM/LGG | Upregulated | Male cancer cell lines (T98G) and female cancer cell lines (LN229) *vs.* male normal cell lines (SVG p12) | Tumor tissues from 36 glioma patients *vs.* paracancerous tissues | ^[13]^ |
|  | GC | Downregulated | — | Tumor tissues from 50 GC patients *vs.* paracancerous tissues | ^[14]^ |
|  | HCC | Upregulated | — | Serum samples from 66 patients with HBV-related HCC *vs.* serum samples from 12 hepatic hemangioma patients; tumor tissues from 66 patients with HBV-related HCC *vs.* non-cancerous liver tissues from hepatic hemangioma patients | ^[3]^ |
|  |  | Upregulated | — | Tumor tissues from 50 HCC patients *vs.* paracancerous tissues | ^[15]^ |
|  |  | Upregulated | Female cancer cell lines (QGY-7703 and Bel-7402) *vs.* female normal cell lines (LO2) | Tumor tissues from 55 HCC patients *vs.* paracancerous tissues | ^[16]^ |
|  |  | Downregulated | Male cancer cell lines (A549 and H1299) and female cancer cell lines (H1915 and HCC827) *vs.* male normal cell lines (16HBE and BEAS-2B) | Tumor tissues from 80 LC patients *vs.* paracancerous tissues | ^[17]^ |
|  | LC | Downregulated | — | Tumor tissues from 15 LC patients *vs.* paracancerous lung tissues | ^[18]^ |
|  |  | Downregulated | Male cancer cell lines [GI-LI-N and SK-N-BE(2)] *vs.* female normal cell lines (HEK293) | Tumor tissues from 30 NB patients *vs.* paracancerous tissues | ^[19]^ |
|  | NB | Downregulated | Male cancer cell lines (A549 and H522) *vs.* male normal cell lines (BEAS-2B) | Tumor tissues from 31 NSCLC patients *vs.* paracancerous tissues | ^[20]^ |
|  | NSCLC | Downregulated | — | Tumor tissues from 84 NSCLC patients *vs.* paracancerous tissues | ^[21]^ |
|  |  | Downregulated | — | Tumor tissues from 43 NSCLC patients *vs.* paracancerous tissues | ^[22]^ |
|  |  | Downregulated | Male cancer cell lines (NCI-H1650) and female cancer cell lines (HCC827) *vs.* male normal cell lines (BEAS-2B) | Tumor tissues from 32 NSCLC patients *vs.* paracancerous tissues | ^[23]^ |
|  |  | Downregulated | — | Tumor tissues from 50 NSCLC patients *vs.* paracancerous tissues | ^[24]^ |
|  |  | Downregulated | Female cancer cell lines (A2780, SKOV3, and ES-2) *vs.* female normal cell lines (IOSE80) | Tumor tissues from 25 OC patients *vs.* paracancerous tissues | ^[25]^ |
|  | OC | Downregulated | Female cancer cell lines (Caov3, OV-90, OVCAR3, and ES-2) *vs.* female normal cell lines (SV40) | Tumor tissues from 60 OC patients *vs.* paracancerous tissues | ^[26]^ |
|  |  | Downregulated | Male cancer cell lines (HSC2, HSC4, and KON) and female cancer cell lines (SAS) *vs.* sex-unspecified normal cell lines (HOK) | Tumor tissues from 20 OSCC patients *vs.* paracancerous tissues | ^[27]^ |
|  | OSCC | Downregulated | — | Tumor tissues from 32 OSCC patients *vs.* paracancerous tissues | ^[28]^ |
|  |  | Downregulated | — | Tumor tissues from 78 PDAC patients *vs.* paracancerous tissues | ^[29]^ |
|  | PDAC | Downregulated | Male cancer cell lines (SW1990 and PANC-1) *vs.* female normal cell lines (HPDE) | Tumor tissues from 41 PDAC patients *vs.* paracancerous tissues | ^[30]^ |
|  |  | Downregulated | — | Tumor tissues from 46 SaOS patients *vs.* paracancerous tissues | ^[31]^ |
|  | SaOS | Downregulated | — | Tumor tissues from 24 RB patients *vs.* paracancerous tissues | ^[32]^ |
|  | RB | Downregulated | Female cancer cell lines (MDA-MB-231, BT-549, HCC-1937, SKBR3, and MCF-7) *vs.* female normal cell lines (MCF-10A) | Tumor tissues from 30 BC patients *vs.* paracancerous tissues | ^[33]^ |
|  | TNBC | Downregulated | Female cancer cell lines (MDA-MB-231 and BT-549) *vs.* female normal cell lines (MCF-10A) | — | ^[34]^ |
|  |  | Downregulated | — | Tumor tissues from 71 CRC patients *vs.* paracancerous tissues | ^[35]^ |
| 5p | CRC | Downregulated | — | Tumor tissues from 30 GC patients *vs.* paracancerous tissues | ^[36]^ |
|  | GC | Downregulated | — | Tumor tissues from 40 LC patients *vs.* paracancerous tissues | ^[37]^ |
|  | LC | Downregulated | Male cancer cell lines (HNE-1) and female cancer cell lines (CNE-2Z, SPC-A1, and 5-8F) *vs.* male normal cell lines (NP69) | Tumor tissues from 32 NPC patients *vs.* paracancerous tissues | ^[38]^ |
|  | NPC | Downregulated | Male cancer cell lines (A549, H1650, and H1299) *vs.* male normal cell lines (16HBE) | Tumor tissues from 30 NSCLC patients *vs.* paracancerous tissues | ^[39]^ |
|  | NSCLC | Downregulated | Female cancer cell lines (SK-OV-3, Caov3, Ov90, and A2780) *vs.* female normal cell lines (HOSE) | Tumor tissues from 36 OC patients *vs.* paracancerous tissues | ^[40]^ |
|  | OC | Downregulated | Male cancer cell lines (SCC-9, SCC-15, SCC-25, and FADU) *vs.* sex-unspecified normal cell lines (HOK) | Tumor tissues from 15 OSCC patients *vs.* paracancerous tissues | ^[41]^ |
|  | OSCC | Downregulated | Male cancer cell lines (MG63) and female cell lines (Saos‑2 and U2OS) *vs.* female normal cell lines (hFOB1.19) | Tumor tissues from 40 SaOS patients *vs.* paracancerous tissues | ^[42]^ |
|  | SaOS |  |  |  |  |

5-FU:5-Fluorouracil ;BCa: Bladder cancer; CRC: Colorectal cancer; EC: Endometrial cancer; GBM/LGG: Glioblastoma/lower-grade glioma; GC: Gastric cancer; HBV: Hepatitis B virus; HCC: Hepatocellular carcinoma; LC: Lung cancer; NB: Neuroblastoma; NPC: Nasopharyngeal carcinoma; NSCLC: Non-small-cell lung cancer; OC: Ovarian cancer; OSCC: Oral squamous cell carcinoma; PDAC: Pancreatic ductal adenocarcinoma; RB: Retinoblastoma; SaOS: Osteosarcoma; TNBC: Triple-negative breast cancer.

**Supplementary** **Table 2: Comparison of TCGA data analysis and existing research on miR-545.**

| **Mature miR-545** | **TCGA cancer** | **Sample size** | **Expression** | **Existing research** |
| --- | --- | --- | --- | --- |
| **miR-545-3p** | ACC | Tumor = 25 | — | Not studied |
|  | BLCA | Tumor = 146, Normal = 1 | Ns | Reduced in BCa tissues |
|  | BRCA | Tumor = 148, Normal = 7 | Ns | Decreased in TNBC cell lines MDA-MB-231, BT-549, HCC-1937, SKBR3, and MCF-7.^33,34^ |
|  | CESC | Tumor = 109, Normal = 2 | Upregulated | Not studied |
|  | CHOL | Tumor = 10, Normal = 2 | Ns | Not studied |
|  | COAD | Tumor = 59 | — | Reduced in CRC tissues and, specifically in the cell lines HCT-8, HCT-116, DLD-1, and SW480.^7,8^ However, the expression levels of miR-545-3p are increased in male CRC cell lines HCT-116, HT-29, and LoVo.^10^ |
|  | DLBC | Tumor = 22 | — | Not studied |
|  | ESCA | Tumor = 51, Normal = 3 | Ns | Not studied |
|  | GBM/LGG | Tumor = 204, Normal = 2 | Downregulated | Increased in GBM/LGG tissues and cells, specifically in the male cell line T98G and the female cell line LN229.^13^ |
|  | HNSC | Tumor = 152, Normal = 6 | Ns | Not studied |
|  | KICH | Tumor = 5, Normal = 7 | Ns | Not studied |
|  | KIRC | Tumor = 49, Normal = 12 | Ns | Not studied |
|  | KIRP | Tumor = 78, Normal = 15 | Upregulated | Not studied |
|  | LIHC | Tumor = 135, Normal = 17 | Ns | Increased in HCC tissues and cells, specifically in the Hep-G2, Hep-3B, and Huh7 cell lines for males and the SMMC-7721, QGY-7703, Bel-703, and Bel-7402 cell lines for females.^15,16^ Additionally, the miR-545-3p expression levels also increased in the serum of HCC patients.^3^ |
|  | LUAD | Tumor = 139, Normal = 2 | Ns | Reduced in NSCLC tissues and cells, specifically in the male cell lines A549, H522, NCI-H1650, and H1299 and the female cell lines H1915 and HCC827.^[20,22-24,43]^ Additionally, the expression levels of miR-545-3p also decreased in the tumor and serum of NSCLC patients.^[43]^ |
|  | LUSC | Tumor = 137, Normal = 13 | Upregulated |  |
|  | MESO | Tumor = 35 | — | Not studied |
|  | OV | Tumor = 6 | — | Reduced in OC tissues and cells, specifically in the female cell lines A2780, SKOV3, Caov3, OV-90, OVCAR3, and ES-2.^[25,26]^ |
|  | PAAD | Tumor = 51 | — | Downregulated in PDAC tissues.^[29]^ |
|  | PCPG | Tumor = 44, Normal = 1 | Downregulated | Not studied |
|  | PRAD | Tumor = 87, Normal = 4 | Ns | Not studied |
|  | READ | Tumor = 39 | — | Not studied |
|  | SARC | Tumor = 62 | — | Not studied |
|  | SKCM | Tumor = 53 | — | Not studied |
|  | STAD | Tumor = 126, Normal = 7 | Ns | Downregulated in GC tissues.^[14]^ |
|  | TGCT | Tumor = 107 | — | Not studied |
|  | THCA | Tumor = 154, Normal = 29 | Ns | Not studied |
|  | THYM | Tumor = 63 | — | Not studied |
|  | UCEC | Tumor = 166, Normal = 8 | Upregulated | Reduced in EC tissues and cells, specifically in the female cell lines HEC-1B, EEC, and Ishikawa.^[11,12]^ |
|  | UCS | Tumor = 26 | — | Not studied |
|  | UVM | Tumor = 7 | — | Not studied |
| **miR-545-5p** | ACC | Tumor = 72 | — | Not studied |
|  | BLCA | Tumor = 309, Normal = 4 | Ns | Not studied |
|  | BRCA | Tumor = 551, Normal = 38 | — | Not studied |
|  | CESC | Tumor = 218, Normal = 2 | Upregulated | Not studied |
|  | CHOL | Tumor = 26, Normal = 6 | Ns | Not studied |
|  | COAD | Tumor = 162 | — | Not studied |
|  | DLBC | Tumor = 44 | — | Not studied |
|  | ESCA | Tumor = 118, Normal = 4 | Ns | Not studied |
|  | GBM/LGG | Tumor = 408, Normal = 3 | Downregulated | Not studied |
|  | HNSC | Tumor = 352, Normal = 22 | Upregulated | Not studied |
|  | KICH | Tumor = 28, Normal = 15 | Ns | Not studied |
|  | KIRC | Tumor = 197, Normal = 49 | Upregulated | Not studied |
|  | KIRP | Tumor = 201, Normal = 24 | Upregulated | Not studied |
|  | LIHC | Tumor = 296, Normal = 41 | Ns | Not studied |
|  | LUAD | Tumor = 310, Normal = 14 | Upregulated | Downregulated in NSCLC cells, specifically in male cell lines A549, H1650, and H1299.^[39]^ |
|  | LUSC | Tumor = 264, Normal = 23 | Upregulated |  |
|  | MESO | Tumor = 70 | — | Not studied |
|  | OV | Tumor = 10 | — | Not studied |
|  | PAAD | Tumor = 103, Normal = 3 | Downregulated | Not studied |
|  | PCPG | Tumor = 127, Normal = 1 | Ns | Not studied |
|  | PRAD | Tumor = 226, Normal = 22 | Ns | Not studied |
|  | READ | Tumor = 68 | — | Not studied |
|  | SARC | Tumor = 151 | — | Not studied |
|  | SKCM | Tumor = 240 | — | Not studied |
|  | STAD | Tumor = 241, Normal = 24 | Ns | Downregulated in GC tissues.^[36]^ |
|  | TGCT | Tumor = 147 | — | Not studied |
|  | THCA | Tumor = 399, Normal = 50 | Ns | Not studied |
|  | THYM | Tumor = 112, Normal = 2 | Ns | Not studied |
|  | UCEC | Tumor = 323, Normal = 24 | Upregulated | Not studied |
|  | UCS | Tumor = 51 | — | Not studied |
|  | UVM | Tumor = 40 | — | Not studied |

Please check the following link for the full name of TCGA abbreviations.

Link: <https://gdc.cancer.gov/resources-tcga-users/tcga-code-tables/tcga-study-abbreviations>.

ACC: Adrenocortical carcinoma; BCa: Bladder cancer; CRC: Colorectal cancer; DLBC: Lymphoid neoplasm diffuse large B-cell lymphoma; GBM/LGG: Glioblastoma/lower-grade glioma; GC: Gastric cancer; HCC: Hepatocellular carcinoma; LIHC: Liver hepatocellular carcinoma; LUSC: Lung squamous cell carcinoma;Ns: Not significant; NSCLC: Non-small cell lung cancer; OC: Ovarian cancer; PCPG: Pheochromocytoma and paraganglioma; PDAC: Pancreatic ductal adenocarcinoma; PRAD: Prostate adenocarcinoma; THCA: Thyroid carcinoma; TNBC: Triple-negative breast cancer; UCS: Uterine carcinosarcoma.

**Supplementary** **Table 3: miR-545–related competitive endogenous RNA (ceRNA) axes in cancer.**

| **Origin of mature miR-545** | **ceRNA/miR-545/PCG axis** | **Cancer** | **Binding site of ceRNA and miR-545** | | **Binding site of miR-545 and PCG** | | **Ref.** |
| --- | --- | --- | --- | --- | --- | --- | --- |
|  |  |  | **ceRNA (5′-…-3′)** | **miR-545 (3′-…-5′)** | **PCG (5′-…-3′)** | **miR-545 (3′-…-5′)** |  |
| 3p | Circ_0001367/miR-545/LUZP1 | GBM/LGG | AAUccAgcGcUUUGCUGA | UUAuUuaCAAACGAC | AUgcccuUUUGCUG | UAuuuacAAACGAC | ^[13]^ |
|  | Circ_0003732/miR-545/CCNA2 | SaOS | CgCAggcAAUGUUGCUGA | GuGUuauUUACaAACGACU | ACuggAucaaUUUGCUGA | UGuuaUuuacAAACGACU | ^[31]^ |
|  | Circ_0067934/miR-545/PPA1 | OC | CuCAAacAggaUUUGCUG | GuGUUauUuacAAACGAC | AuaaAAUuAUUUUGCUG | UgugUuAUuuACAAACGAC | ^[25]^ |
|  | Circ_0072088/miR-545/SLC7A11 | PDAC | UUUGCUG | AAACGAC | UGUUUGCUG | ACAAACGAC | ^[30]^ |
|  | Circ_0007580/miR-545/PRKCA | NSCLC | AUAucaaaaUUGCUG | UAUuuacaAACGAC | — | — | ^[44]^ |
|  | Circ_0014130/miR-545/YAP1 | NSCLC | UUUGCUG | AAACGAC | UUGCUG | AACGAC | ^[22]^ |
|  | Circ_0026416/miR-545/MYO6 | CRC | UUGCUG | AACGAC | UUUGCUG | AAACGAC | ^[7]^ |
|  | Circ_0067934/miR-545/EIF3C | CxCa | UUUGCUGA | AAACGACU | UUUGCUGA | AAACGACU | ^[45]^ |
|  | Circ_0067934/miR-545/SLC7A11 | TC | UUUGCUG | AAACGAC | AaAaAuUAguUGUUUGCUG | UgUgUuAUuuACAAACGAC | ^[46]^ |
|  | Circ_0072083/miR-545/CBLL1 | NSCLC | AucuAAUcAAGUUUGCUG | UgugUUAuUUaCAAACGAC | UUUGCUG | AAACGAC | ^[20]^ |
|  | Circ_FGGY/miR-545/SMAD7 | HCC | UUUGCUG | AAACGAC | UUUGCUG | AAACGAC | ^[15]^ |
|  | Circ_FOXO3/miR-545/HMGB3 | NSCLC | AucCgAUgAugUccUUUGCUG | UguGuUAuUuAcAAACGAC | ACuCugUgcAcUUUGCUG | UGuGuuAuuUacAAACGAC | ^[24]^ |
|  | Circ_IFT80/miR-545/FAM98A | EC | UUUGCUG | AAACGAC | UUGCUG | AACGAC | ^[11]^ |
|  | Circ_PRKCI/miR-545 | GC | UUUGCUG | AAACGAC | — | — | ^[14]^ |
|  | Circ_PRKCI/miR-545/WBP2 | TNBC | UUUGCUGA | AAACGACU | UUUGCUG; UUUGCUGA | AAACGAC; AAACGACU | ^[33]^ |
|  | Circ_ SAMD4A/miR-545/ PFKFB3 | CRC | UUUGCUG | AAACGAC | UUGCUG | AACGAC | ^[47]^ |
|  | Circ_ZFR/miR-545/WMT5A | BCa | UUUGCUG | AAACGAC | — | — | ^[6]^ |
|  | AFAP1-AS1/miR-545/CDK4 | TNBC | UaUuUgAGGUUUG | GuUaUuUACAAAC | UACUUU | UACAAA | ^[34]^ |
|  | AFAP1-AS1/miR-545/HDGF | LC | AuUugAgGUUUGCUG | UUAuUuaCAAACGAC | AUgAAaaaUUGCUG | UAuUUacaAACGAC | ^[17]^ |
|  | AFAP1-AS1/miR-545/VEGFA | EC | UUUGCUG | AAACGAC | UUGCUG | AACGAC | ^[12]^ |
|  | AFAP1-AS1/miR-545/GNB1 | RB | uAuUugAgGUUUGCUG | gUuAuuUaCAAACGAC | UUUGCUGA; UUGCUGA | AAACGACU; AACGACU | ^[32]^ |
|  | CASC9/miR-545/LAMC2 | OSCC | CAgAuggAcAcaUUUGCUG | GUgUuaUuUacAAACGAC | UUGCUG | AACGAC | ^[28]^ |
|  | FAM83H-AS1/miR-545/HS6ST2 | NSCLC | UUUGCUG; UUUGCUGA | AAACGAC; AAACGACU | UUGCUGA | AACGACU | ^[23]^ |
|  | HOTAIR/miR-545/EGFR | LC | — | — | AAUAUUUGCUG | UUAUuuacAAACGAC | ^[8]^ |
|  | LINC00261/miR-545/MT1M | ESCC | CACAAgcUgggcGUUUGCUG | GUgUuaUuUacAAACGAC | UUUGCUG | AAACGAC | ^[48]^ |
|  | LINC01410/miR-545/HK2 | NB | AAgUGUUGCUGA | UUuACaAACGACU | UUGCUG | AACGAC | ^[19]^ |
|  | MCM3AP-AS1/miR-545/CDK4 | CRC | GuAugggAaAAuUGUUUGC | CgUguguUaUUuACAAACG | — | — | ^[49]^ |
|  | SBF2-AS1/miR-545/EMS1 | GC | UUGCUGA | AACGACU | — | — | ^[50]^ |
|  | miR-545/ARGLU | HCC | — | — | UUUGCUG | AAACGAC | ^[3]^ |
|  | miR-545/CCND1 | LC | — | — | UUUGCUG | AAACGAC | ^[18]^ |
|  | miR-545/CDK4 | LC | — | — | UUUGCUG | AAACGAC | ^[18]^ |
|  | miR-545/ESRRA | HCC | — | — | UUUGCUG | AAACGAC | ^[3]^ |
|  | miR-545/ESRRG | HCC | — | — | UUUGCUG | AAACGAC | ^[3]^ |
|  | miR-545/KDM4B | OC | — | — | UUGCUG | AACGAC | ^[26]^ |
|  | miR-545/KU70 | LC | — | — | UUUGCUG | AAACGAC | ^[51]^ |
|  | miR-545/MT1M | HCC | — | — | UGAGCCA | ACUCGGU | ^[16]^ |
|  | miR-545/PLK1 | OC | — | — | UUGCUG | AACGAC | ^[26]^ |
|  | miR-545/RIG-I | CRC | — | — | UUUGCUG | AAACGAC | ^[9]^ |
|  | miR-545/RIG-I | OSCC | — | — | UUUGCUG | AAACGAC | ^[27]^ |
|  | miR-545/RIG-I | PDAC | — | — | UUUGCUG | AAACGAC | ^[29]^ |
|  | miR-545/TF | CRC | — | — | GUCGAAC | ACGACUA | ^[10]^ |
|  | miR-545/ZEB2 | NSCLC | — | — | UUUGCUG | AAACGAC | ^[21]^ |
|  | Circ_UBR1/miR-545/SSFA2 | LC | UCaguUcAgAugUUUACUG | AGauuAUuUguAAAUGAC | UCUAugAgguAUUUACUG | AGAUuaUuugUAAAUGAC | ^[37]^ |
| 5p | CRNDE/miR-545/CCND2 | NPC | uAUuUgAUugcCAguUUUACUGA | gUAgAuUAuuuGUAAAUGACU | — | — | ^[38]^ |
|  | CRNDE/miR-545/TIM-3 | OSCC | UUUACUGA | AAAUGACU | UUUGCUG | AAAUGAC | ^[41]^ |
|  | LINC00342/miR-545/CNPY2 | GC | AgAUUUACUG; AUuUgAcUcucugcUUUACUG | UgUAAAUGAC; UAgAuUAuuuguAAAUGAC | AUaUAuUAuguuUUUACUG | UAgAUuAUuuguAAAUGAC | ^[36]^ |
|  | LINC00342/miR-545/MDM2 | CRC | AgAUUUACUG | UgUAAAUGAC | UUUGCUG | AAACGAC | ^[35]^ |
|  | LncRP5/miR-545/PTP4A1 | OC | CAUUUACAuAgggUgUAC | GUAAAUGUaUauuAgAUG | UUACUG | AAUGAC | ^[40]^ |
|  | NR2F2-AS1/miR-545/c-Met | NSCLC | UCAgCgcacUAAAuAUUUACUGA | AGUaGauuAUUUgUAAAUGACU | UUACUG | AAUGAC | ^[39]^ |
|  | miR-545/DIMT1 | SaOS | — | — | UUUGCUG | AAAUGAC | ^[42]^ |
|  |  |  |  |  |  |  |  |

CRC: Colorectal cancer; EC: Endometrial cancer; ESCC: Esophageal squamous cell carcinoma; GBM/LGG: Glioblastoma/lower-grade glioma; GC: Gastric cancer; HCC: Hepatocellular carcinoma; LC; Lung cancer; NB: Neuroblastoma; NPC: Nasopharyngeal carcinoma; NSCLC: Non-small cell lung cancer; OC: Ovarian cancer; OSCC: Oral squamous cell carcinoma; PDAC: Pancreatic ductal adenocarcinoma; RB: Retinoblastoma; SaOS: Osteosarcoma; TNBC: Triple-negative breast cancer.

**Supplementary** **Table 4: Effects of miR-545 *in vivo* and *in vitro.***

| **Origin of mature miR-545** | **System** | **Cancer** | **PCG** | **Effects *in vitro*** | **Related cell line** | **Effects *in vivo*** | **Xenograft model** | **Ref.** |
| --- | --- | --- | --- | --- | --- | --- | --- | --- |
| 3p | Digestive system | CRC | CDK4 | Proliferation↓ and cell cycle↓ | CR4 | — | — | ^[49]^ |
|  |  |  | EGFR | Proliferation↓ and viability↓ | SW480 and LOVO | Tumor growth↓ | LOVO cell xenograft in BALB/c nude mice | ^[8]^ |
|  |  |  | MYO6 | Proliferation↓, colony formation↓, invasion↓, migration↓, and EMT↓ | HCT-8 and SW480 | — | — | ^[7]^ |
|  |  |  | RIG-I | Proliferation↑ | DLD-1 and HCT-116 | — | — | ^[9]^ |
|  |  |  | PFKFB3 | Proliferation↓, apoptosis↑, and 5-FU resistance↓ | SW480/5-FU and HCT-116/5-FU | 5-FU resistance↑ | SW480/5-FU cell xenograft in BALB/c nude mice | ^[47]^ |
|  |  |  | TF | Lipid-oxidation↓ and iron-accumulation↓ | HT-29 and HCT-116 | Tumor growth↑ | HT-29 and HCT-116 cell xenograft in C57BL/6 mice | ^[10]^ |
|  |  | ESCC | MT1M | Proliferation↑, apoptosis↓, and DDP resistance↑ | TE-1 and ESCC109 | — | — | ^[48]^ |
|  |  | GC | EMS1 | Proliferation↓, invasion↓, and migration↓ | SGC7901 | — | — | ^[50]^ |
|  |  | HCC | MT1M | Invasion↑ and migration↑ | QGY-7703 and SMMC-7721 | Tumor growth↑ | SMMC-7721 and QGY-7703 cell xenograft in BALB/c nude mice | ^[16]^ |
|  |  |  | SMAD7 | Proliferation↑, migration↑, and invasion↑ | HepG2 and MHCC97H | Tumor growth↑ | MHCC97H cell xenograft in male BALB/c nude mice | ^[15]^ |
|  |  |  | LAMC2 | Proliferation↓, colony formation↓, migration↓, viability↓, and cell cycle↓ | SCC4 and SCC9 | — | — | ^[28]^ |
|  |  | OSCC | RIG-I | Proliferation↓, and migration↓ | HSC4 | — | — | ^[27]^ |
|  |  |  | SLC7A11 | Proliferation↓, migration↓, invasion↓, and glycolysis↓ | SW1990 and PANC-1 | Tumor growth↓ | PANC-1 cell xenograft in male BALB/c nude mice | ^[30]^ |
|  |  | PDAC | RIG-I | Proliferation↓ | HEK293, PANC1, and SW1990 | — | — | ^[29]^ |
|  |  |  | SLC7A11 | Ferroptosis↑, apoptosis↑, and viability↓ | FTC133 and TPC-1 | — | — | ^[46]^ |
|  | Endocrine system | TC | CCNA2 | Proliferation↓ | MG-63 | — | — | ^[31]^ |
|  | Motor system | SaOS | LUZP1 | Proliferation↑, invasion↑, and migration↑ | LN229 and T98G | — | — | ^[13]^ |
|  | Nervous system | GBM/LGG | HK2 | Viability↓, colony formation↓, invasion↓, and radioresistance↓ | GI-LI-N and SK-N-BE(2) | — | — | ^[19]^ |
|  |  | NB | GNB1 | Proliferation↓ and migration↓ | Y79 and WERI-Rb-1 | Tumor growth↓ | WERI-Rb-1 cell xenograft in female BALB/c nude mice | ^[32]^ |
|  |  | RB | EIF3C | Proliferation↓, invasion↓, and migration↓ | SiHa and Hela | — | — | ^[45]^ |
|  | Reproductive system | CxCa | FAM98A | Proliferation↓ and apoptosis↑ | HEC-1B and Ishikawa | — | — | ^[11]^ |
|  |  | EC | VEGFA | Proliferation↓, invasion↓, migration↓, and angiogenesis↓ | Ishikawa and HEC1-A | — | — | ^[12]^ |
|  |  |  | PPA1 | Proliferation↓, invasion↓, apoptosis↑, and DDP resistance↓ | A2780/DDP | Tumorigenesis↓ | A2780/DDP cell xenograft in female BALB/c nude mice | ^[25]^ |
|  |  | OC | KDM4B and PLK1 | Invasion↓, migration↓ and angiogenesis↓ | OV-90 and ES-2 | Tumor growth↓ and metastasis↓ | OV-90 and ES-2 cell xenograft in BALB/c nude mice | ^[26]^ |
|  |  |  | WBP2 | Proliferation↓ and invasion↓ | MDA-MB-231 and BT-549 | Tumor growth↓ | MDA-MB-231 cell xenograft in female BALB/c nude mice | ^[33]^ |
|  |  | TNBC | CDK4 | Proliferation↓, invasion↓, migration↓, ADM resistance↓, DTX resistance↓, PTX resistance↓, and DDP resistance↓ | MDA-MB-231 and BT-549 | — | — | ^[34]^ |
|  |  |  | CCND1 and CDK4 | Viability↓, proliferation↓, and cell cycle↓ | A549, H460, and HFL1 | Tumor growth↓ | A549 cell xenograft in nude mice | ^[18]^ |
|  | Respiratory system | LC | HDGF | Proliferation↓, invasion↓, migration↓, and apoptosis↑ | A549 | — | — | ^[17]^ |
|  |  |  | Ku70 | Proliferation↓ and apoptosis↑ | LLC cell | Radiosensitivity↑ | LLC cell xenograft in C57BL/6 mice | ^[51]^ |
|  |  |  | CBLL1 | Cell cycle↓, colony formation↓, metastasis↓, apoptosis↑, and EMT↓ | A549 and H522 | — | — | ^[20]^ |
|  |  | NSCLC | HMGB3 | Proliferation↓, invasion↓, and migration↓ | A549 and H1229 | — | — | ^[24]^ |
|  |  |  | PRKCA | Invasion↓ and apoptosis↑ | A549 | — | — | ^[44]^ |
|  |  |  | YAP1 | Colony formation↓, invasion↓, migration↓, apoptosis↑, and DTX resistance↓ | NCI-H1299 and A549 | — | — | ^[22]^ |
|  |  |  | HS6ST2 | Proliferation↓ and invasion↓ | NCI-H1650 and HCC827 | Tumor growth↓ | HCC827 cell xenograft in female BALB/c nude mice | ^[23]^ |
|  |  |  | ZEB2 | Proliferation↓, invasion↓, and migration↓ | A549 | — | — | ^[21]^ |
|  |  |  | MDM2 | Proliferation↓, invasion↓, migration↓, and apoptosis↑ | HCT-116 and SW620 | — | — | ^[35]^ |
| 5p | Digestive system | CRC | CNPY2 | Proliferation↓, invasion↓, and migration↓ | AGS | — | — | ^[36]^ |
|  |  | GC | TIM-3 | Immune response↑ | CD8+ T cell | — | — | ^[41]^ |
|  |  | OSCC | PTP4A1 | Proliferation↓ and metastasis↓ | SKOV3 and A2780 | Tumor growth↓ | A2780 cell xenograft in female BALB/cA nude mice | ^[40]^ |
|  | Reproductive system | OC | DIMT1 | Proliferation↓, invasion↓, and migration↓ | Saos‑2 and MG63 | Tumor growth↓ | Saos‑2 and MG63 cell xenograft in BALB/c nude mice | ^[42]^ |
|  | Motor system | SaOS | SSFA2 | Proliferation↓, invasion↓, migration↓, and apoptosis↑ | A549 | — | — | ^[37]^ |
|  | Respiratory system | LC | CCND2 | Cell cycle↓, proliferation↓, invasion↓, migration↓, apoptosis↑, and EMT↓ | CNE-2Z and HNE-1 | — | — | ^[38]^ |
|  |  | NPC | c-Met | Proliferation↓, invasion↓, migration↓, and EMT↓ | A549 | Tumor growth↓ | A549 cell xenograft in BALB/c mice | ^[39]^ |
|  |  | NSCLC |  |  |  |  |  |  |

5-FU: 5-fluorouracil; ADM: Adriamycin; CRC: Colorectal cancer; DDP: Cisplatin; DTX: Docetaxel; EC: Endometrial cancer; EMT: Epithelial-mesenchymal transition; ESCC: Esophageal squamous cell carcinoma; GBM/LGG: Glioblastoma/lower-grade glioma; GC: Gastric cancer; HCC: Hepatocellular carcinoma; LC: Lung cancer; LLC: Lewis lung carcinoma; NB: Neuroblastoma; NPC: Nasopharyngeal carcinoma; NSCLC: Non-small-cell lung cancer; OC: Ovarian cancer; OSCC: Oral squamous cell carcinoma; PDAC: Pancreatic ductal adenocarcinoma; PTX: Paclitaxel; RB: Retinoblastoma; SaOS: Osteosarcoma; TC: Thyroid carcinoma; TF: Transferrin; TNBC: Triple-negative breast cancer.

**Supplementary Figure Legends**


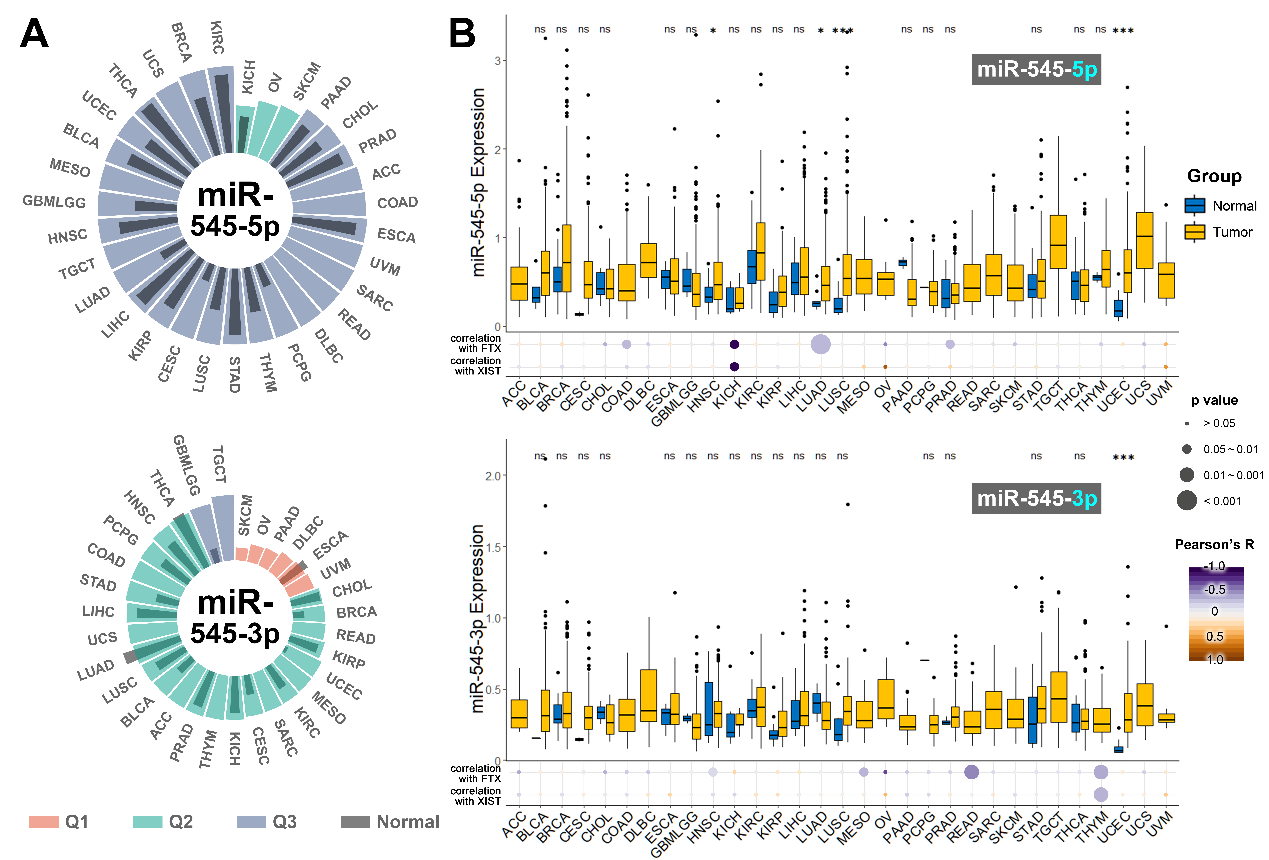


**Supplementary** **Figure 1.** Pan-cancer analysis of miR-545. (A) Relative miR-545 expression levels in each cancer type, calculated as the expression ranking divided by the number of miRNAs with non-zero expression. (B) miR-545 expression levels in each cancer type, calculated as log2(RPM+1). The "***", "**", and "*" indicate an adjusted *P* value < 0.001, 0.01, and 0.05, respectively; "ns" indicates no significant difference. Please check the following link for the full name of The Cancer Genome Atlas (TCGA) abbreviations. Link: <https://gdc.cancer.gov/resources-tcga-users/tcga-code-tables/tcga-study-abbreviations>.


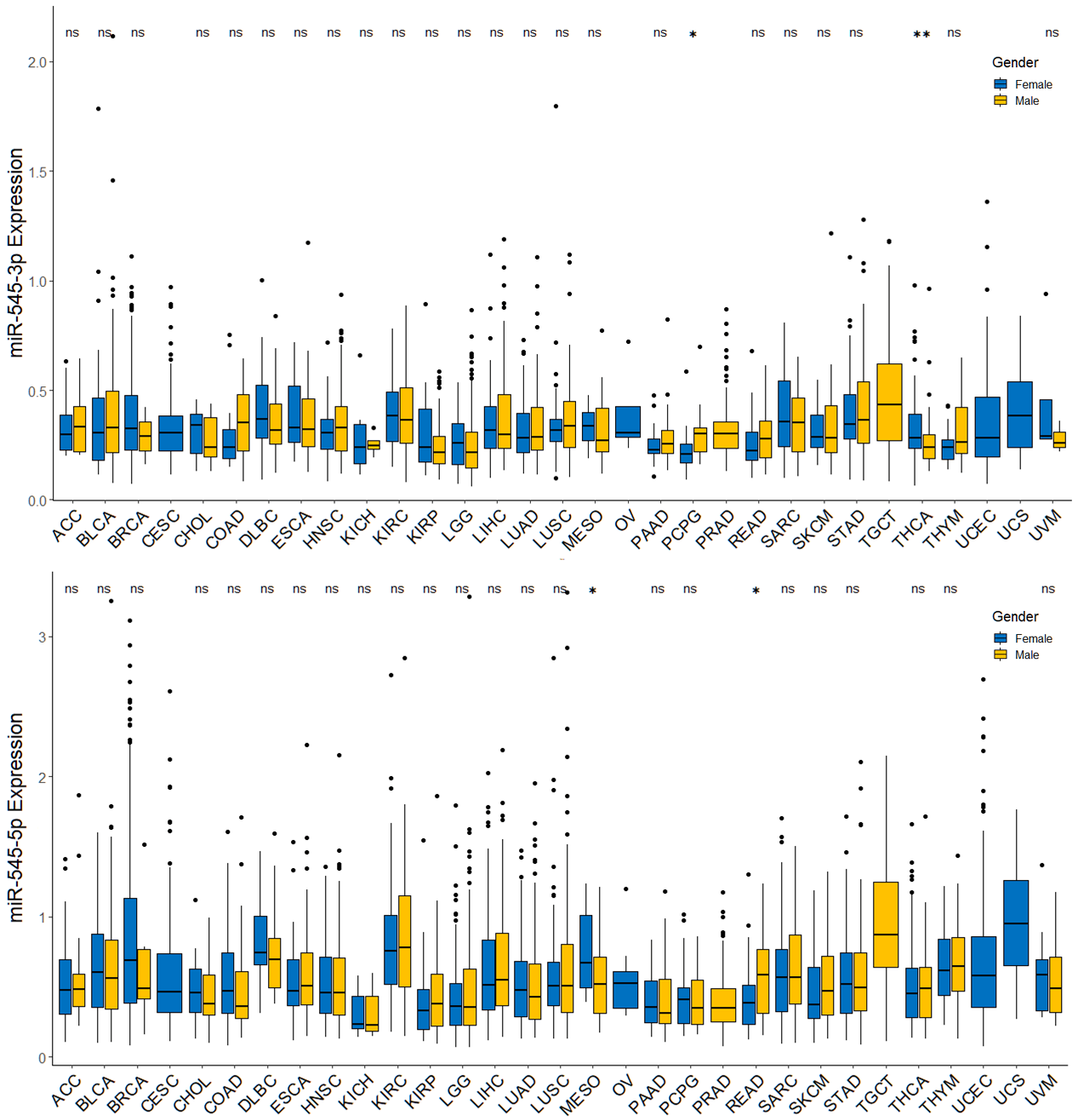


**Supplementary** **Figure 2.** miR-545 expression differences between males and females. An asterisk (*) denotes an adjusted *P* value < 0.05, whereas ‘ns’ indicates no significant difference. There was no significant difference in miR-545 expression between sexes in most cancer types. However, in PCPG, miR-545-3p expression was significantly higher in males, whereas in THCA it was higher in females. Similarly, miR-545-5p expression was significantly higher in females with MESO and males with READ (*P* < 0.05). Please check the following link for the full names of The Cancer Genome Atlas abbreviations. Link: <https://gdc.cancer.gov/resources-tcga-users/tcga-code-tables/tcga-study-abbreviations>. MESO:Mesothelioma ;THCA:Thyroid carcinoma ;READ:Rectum adenocarcinoma;


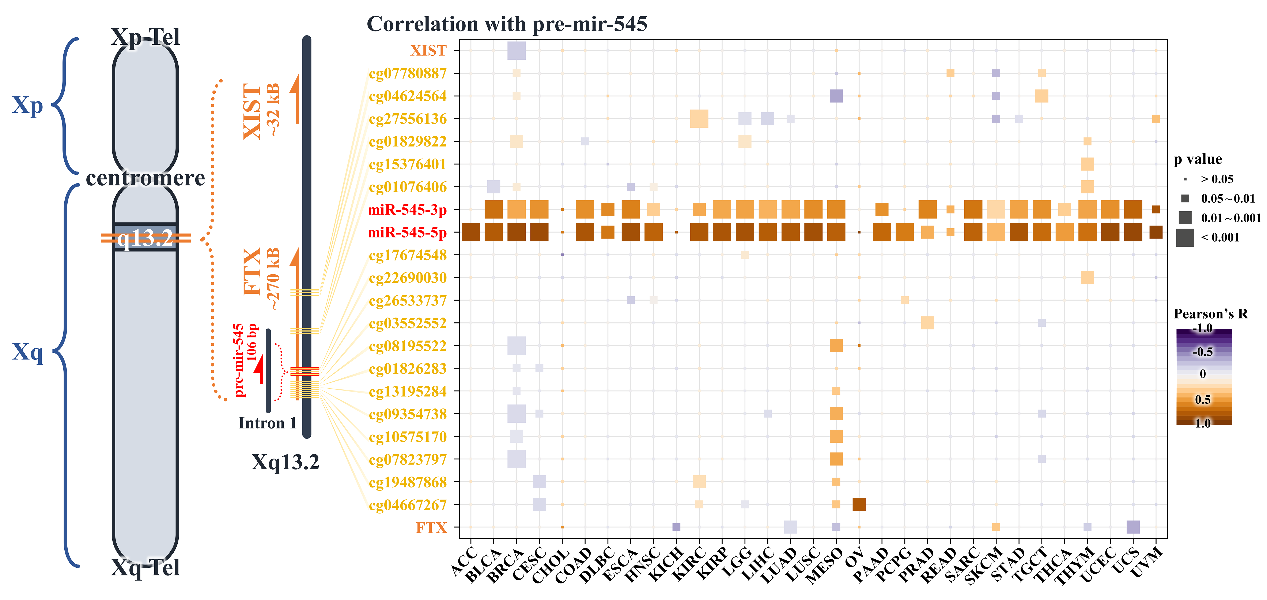


**Supplementary** **Figure 3.** Chromosomal location of pre-miR-545 and its host gene *FTX*. Pre-miR-545 is located in the Xq13.2 region, and it was significantly positively correlated with miR-545-3p in all cancers except CHOL, KICH, OV, and PCPG. It was also significantly and positively correlated with miR-545-5p in all cancers except CHOL, KICH, and OV. Pre-miR-545 was significantly negatively correlated with *XIST* in BRCA and *FTX* in KICH, LUAD, MESO, THYM, and UCS. It was also significantly negatively correlated with *FTX* levels in the SKCM group (*P* < 0.05, absolute value of *r* > 0.3). Please check the following link for the full names of The Cancer Genome Atlas abbreviations. Link: <https://gdc.cancer.gov/resources-tcga-users/tcga-code-tables/tcga-study-abbreviations>.


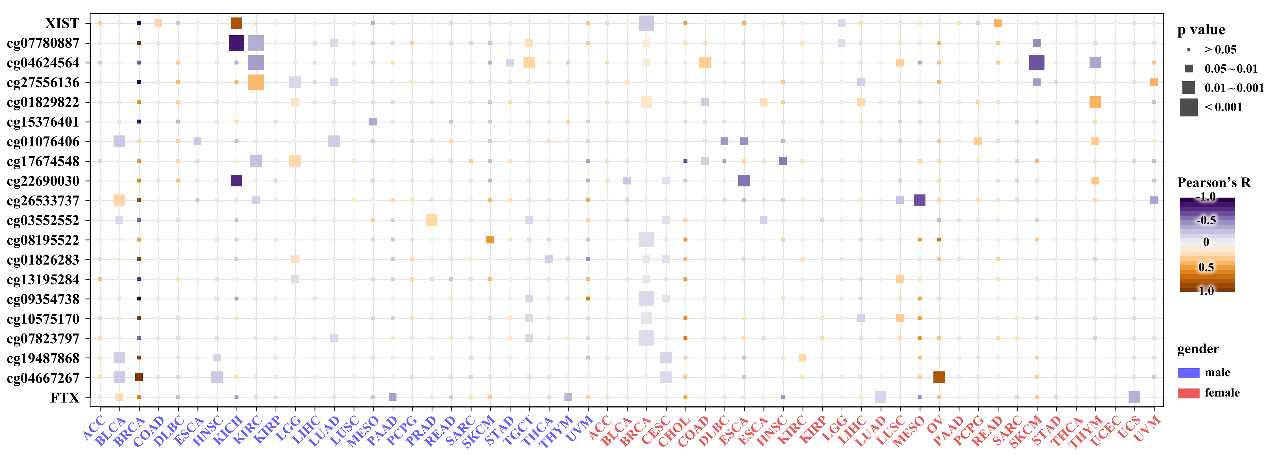


**Supplementary** **Figure 4.** Correlation between pre-miR-545 expression levels and *FTX* in different sexes**.** Please check the following link for the full name of The Cancer Genome Atlas abbreviations. Link: <https://gdc.cancer.gov/resources-tcga-users/tcga-code-tables/tcga-study-abbreviations>.


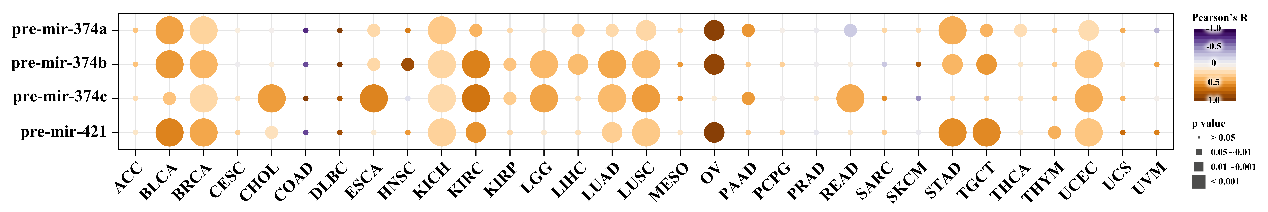


**Supplementary** **Figure 5.** Correlation between pre-miR-545 and other miRNA clusters in *FTX*. Please check the following link for the full name of The Cancer Genome Atlas abbreviations. Link: <https://gdc.cancer.gov/resources-tcga-users/tcga-code-tables/tcga-study-abbreviations>.

**References**

1. Cancer Genome Atlas Research Network, Weinstein JN, Collisson EA, Mills GB, Shaw KR, Ozenberger BA, *et al*. The Cancer Genome Atlas Pan-Cancer analysis project. Nat Genet 2013;45:1113–1120. doi: 10.1038/ng.2764.

2. Goldman MJ, Craft B, Hastie M, Repečka K, McDade F, Kamath A, *et al*. Visualizing and interpreting cancer genomics data via the Xena platform. Nat Biotechnol 2020;38:675–678. doi: 10.1038/s41587-020-0546-8.

3. Zhao Q, Li T, Qi J, Liu J, Qin C. The miR-545/374a cluster encoded in the *FTX* lncRNA is overexpressed in HBV-related hepatocellular carcinoma and promotes tumorigenesis and tumor progression. PLoS ONE 2014;9:e109782. doi: 10.1371/journal.pone.0109782.

4. Kozomara A, Griffiths-Jones S. miRBase: Integrating microRNA annotation and deep-sequencing data. Nucleic Acids Res 2011;39:D152–D157. doi: 10.1093/nar/gkq1027.

5. Cain JA, Montibus B, Oakey RJ. Intragenic CpG islands and their impact on gene regulation. Front Cell Dev Biol 2022;10:832348. doi: 10.3389/fcell.2022.832348.

6. Luo L, Miao P, Ming Y, Tao J, Shen H. Circ-ZFR promotes progression of bladder cancer by upregulating WNT5A via sponging miR-545 and miR-1270. Front Oncol 2020;10:596623. doi: 10.3389/fonc.2020.596623.

7. Zhang L, Yu R, Li C, Dang Y, Yi X, Wang L. Circ_0026416 downregulation blocks the development of colorectal cancer through depleting MYO6 expression by enriching miR-545-3p. World J Surg Oncol 2021;19:299. doi: 10.1186/s12957-021-02407-y.

8. Huang X, Lu S. MicroR-545 mediates colorectal cancer cells proliferation through up-regulating epidermal growth factor receptor expression in HOTAIR long non-coding RNA dependent. Mol Cell Biochem 2017;431:45–54. doi: 10.1007/s11010-017-2974-4.

9. Kwok ZH, Zhang B, Chew XH, Chan JJ, Teh V, Yang H, *et al*. Systematic analysis of intronic miRNAs reveals cooperativity within the multicomponent *FTX* locus to promote colon cancer development. Cancer Res 2021;81:1308–1320. doi: 10.1158/0008-5472.CAN-20-1406.

10. Zheng S, Hu L, Song Q, Shan Y, Yin G, Zhu H, *et al*. miR-545 promotes colorectal cancer by inhibiting transferring in the non-normal ferroptosis signaling. Aging (Albany NY) 2021;13:26137–26147. doi: 10.18632/aging.203801.

11. Wang N, Guo Y, Song L, Tong T, Fan X. Circular RNA intraflagellar transport 80 facilitates endometrial cancer progression through modulating miR-545-3p/FAM98A signaling. J Gynecol Oncol 2022;33:e2. doi: 10.3802/jgo.2022.33.e2.

12. Zhong Y, Wang Y, Dang H, Wu X. LncRNA AFAP1-AS1 contributes to the progression of endometrial carcinoma by regulating miR-545-3p/VEGFA pathway. Mol Cell Probes 2020;53:101606. doi: 10.1016/j.mcp.2020.101606.

13. Dong X, Zhang P, Liu L, Li H, Cheng S, Li S, *et al*. The Circ_0001367/miR-545-3p/LUZP1 axis regulates cell proliferation, migration and invasion in glioma cells. Front Oncol 2021;11:781471. doi: 10.3389/fonc.2021.781471.

14. Wu L, Li Y, Xu XM, Zhu X. Circular RNA circ-PRKCI promotes cell proliferation and invasion by binding to microRNA-545 in gastric cancer. Eur Rev Med Pharmacol Sci 2019;23:9418–9426. doi: 10.26355/eurrev_201911_19435.

15. Feng KL, Diao N, Zhou ZW, Fang CK, Wang JN, Zhang Y, *et al*. CircFGGY inhibits cell growth, invasion and epithelial-mesenchymal transition of hepatocellular carcinoma via regulating the miR-545-3p/Smad7 axis. Front Cell Dev Biol 2022;10:850708. doi: 10.3389/fcell.2022.850708.

16. Changjun L, Feizhou H, Dezhen P, Zhao L, Xianhai M. MiR-545-3p/MT1M axis regulates cell proliferation, invasion and migration in hepatocellular carcinoma. Biomed Pharmacother 2018;108:347–354. doi: 10.1016/j.biopha.2018.09.009.

17. Sun J, Min H, Yu L, Yu G, Shi Y, Sun J. The knockdown of LncRNA AFAP1-AS1 suppressed cell proliferation, migration, and invasion, and promoted apoptosis by regulating miR-545-3p/hepatoma-derived growth factor axis in lung cancer. Anti Cancer Drugs 2021;32:11–21. doi: 10.1097/CAD.0000000000001003.

18. Du B, Wang Z, Zhang X, Feng S, Wang G, He J, *et al*. MicroRNA-545 suppresses cell proliferation by targeting cyclin D1 and CDK4 in lung cancer cells. PLoS ONE 2014;9:e88022. doi: 10.1371/journal.pone.0088022.

19. Mou L, Wang L, Zhang S, Wang Q. Long noncoding RNA LINC01410 suppresses tumorigenesis and enhances radiosensitivity in neuroblastoma cells through regulating miR-545-3p/HK2 axis. Onco Targets Ther 2021;14:3225–3238. doi: 10.2147/OTT.S297969.

20. Li H, Liu F, Qin W. Circ_0072083 interference enhances growth-inhibiting effects of cisplatin in non-small-cell lung cancer cells via miR-545-3p/CBLL1 axis. Cancer Cell Int 2020;20:78. doi: 10.1186/s12935-020-1162-x.

21. Cui J, Pan G, He Q, Yin L, Guo R, Bi H. MicroRNA-545 targets ZEB2 to inhibit the development of non-small cell lung cancer by inactivating Wnt/β-catenin pathway. Oncol Lett 2019;18:2931–2938. doi: 10.3892/ol.2019.10619.

22. Du D, Cao X, Duan X, Zhang X. Blocking circ_0014130 suppressed drug resistance and malignant behaviors of docetaxel resistance-acquired NSCLC cells via regulating miR-545-3p-YAP1 axis. Cytotechnology 2021;73:571–584. doi: 10.1007/s10616-021-00478-z.

23. Zhang Y, Yu Y, Cao X, Chen P. Role of lncRNA FAM83H antisense RNA1 (FAM83H-AS1) in the progression of non-small cell lung cancer by regulating the miR-545-3p/heparan sulfate 6-O-sulfotransferase (HS6ST2) axis. Bioengineered 2022;13:6476–6489. doi: 10.1080/21655979.2022.2031668.

24. Yang M, Zheng E, Ni J, Xu X, Jiang X, Zhao G. Circular RNA circFOXO3 facilitate non-small cell lung cancer progression through upregulating HMGB3 via sponging miR-545-3p/miR-506-3p. Tissue Cell 2022;75:101702. doi: 10.1016/j.tice.2021.101702.

25. Yin Y, Li J, Rong J, Zhang B, Wang X, Han H. Circ_0067934 reduces JNK phosphorylation through a microRNA-545-3p/PPA1 axis to enhance tumorigenesis and cisplatin resistance in ovarian cancer. Immunopharmacol Immunotoxicol 2022;44:261–274. doi: 10.1080/08923973.2022.2038193.

26. Zhang H, Zhang K, Xu Z, Chen Z, Wang Q, Wang C, *et al*. MicroRNA-545 suppresses progression of ovarian cancer through mediating PLK1 expression by a direct binding and an indirect regulation involving KDM4B-mediated demethylation. BMC Cancer 2021;21:163. doi: 10.1186/s12885-021-07830-8.

27. Yuan G, Wu H, Du Y, He F. Tumor suppressor role of microRNA-545 in oral squamous cell carcinoma. Oncol Lett 2019;17:2063–2068. doi: 10.3892/ol.2018.9820.

28. Ning B, Guo S, Mei Y. Long non-coding RNA CASC9 promotes tumor progression in oral squamous cell carcinoma by regulating microRNA-545-3p/laminin subunit gamma 2. Bioengineered 2021;12:7907–7919. doi: 10.1080/21655979.2021.1977103.

29. Song B, Ji W, Guo S, Liu A, Jing W, Shao C, *et al*. miR-545 inhibited pancreatic ductal adenocarcinoma growth by targeting RIG-I. FEBS Lett 2014;588:4375–4381. doi: 10.1016/j.febslet.2014.10.004.

30. Sun H, Liu F, Zhang H. Circ_0072008, an oncogene in pancreatic ductal adenocarcinoma, contributes to tumour cell malignant progression and glycolysis by regulating miR-545-3p/SLC7A11 axis. Autoimmunity 2022;55:203–213. doi: 10.1080/08916934.2022.2027919.

31. Li L, Kong XA, Zang M, Dong J, Feng Y, Gui B, *et al*. Hsa_circ_0003732 promotes osteosarcoma cells proliferation via miR-545/CCNA2 axis. Biosci Rep 2020;40:BSR20191552. doi: 10.1042/BSR20191552.

32. Tang W, Zhang L, Li J, Guan Y. AFAP1 antisense RNA 1 promotes retinoblastoma progression by sponging microRNA miR-545-3p that targets G protein subunit beta 1. Bioengineered 2022;13:5638–5652. doi: 10.1080/21655979.2022.2033464.

33. Wang X, Song H, Fang L, Wu T. EIF4A3-mediated circPRKCI expression promotes triple-negative breast cancer progression by regulating WBP2 and PI3K/AKT signaling pathway. Cell Death Discov 2022;8:92. doi: 10.1038/s41420-022-00892-y.

34. Wu J, Xu W, Ma L, Sheng J, Ye M, Chen H, *et al*. Formononetin relieves the facilitating effect of lncRNA AFAP1-AS1-miR-195/miR-545 axis on progression and chemo-resistance of triple-negative breast cancer. Aging (Albany NY) 2021;13:18191–18222. doi: 10.18632/aging.203156.

35. Miao Z, Liu S, Xiao X, Li D. LINC00342 regulates cell proliferation, apoptosis, migration and invasion in colon adenocarcinoma via miR-545-5p/MDM2 axis. Gene 2020;743:144604. doi: 10.1016/j.gene.2020.144604.

36. Liu R, Yang X, LINC LR. LncRNA LINC00342 promotes gastric cancer progression by targeting the miR-545-5p/CNPY2 axis. BMC Cancer 2021;21:1163. doi: 10.1186/s12885-021-08829-x.

37. Su P, Mao F, Zhang J, Zhang H, Wang M, Xu Y, *et al*. Circular RNA UBR1 promotes the proliferation, migration, and invasion but represses apoptosis of lung cancer cells via modulating microRNA-545-5p/SSFA2 axis. Bioengineered 2021;12:12135–12147. doi: 10.1080/21655979.2021.2004977.

38. Ge S, Jiang C, Li M, Cheng Z, Feng X. Long non-coding RNA CRNDE exacerbates NPC advancement mediated by the miR-545-5p/CCND2 axis. Cancer Cell Int 2021;21:650. doi: 10.1186/s12935-021-02348-2.

39. Liu C, Li QG, Zhou Y, Cao YY, Wei ZX, Jin YH, *et al*. LncRNA NR2F2-AS1 induces epithelial-mesenchymal transition of non-small cell lung cancer by modulating BVR/ATF-2 pathway via regulating miR-545-5p/c-Met axis. Am J Cancer Res 2021;11:4844–4865.

40. Sun P, Bao A, Hua X, Cao J, Ding Y. RP5-1148A21.3 (lncRP5) exerts oncogenic function in human ovarian carcinoma. Acta Biochim Biophys Sin (Shanghai) 2022;54:209–219. doi: 10.3724/abbs.2022002.

41. Ai Y, Wu S, Gao H, Wei H, Tang Z, Li X, *et al*. Repression of CRNDE enhances the anti-tumour activity of CD8 + T cells against oral squamous cell carcinoma through regulating miR-545-5p and TIM-3. J Cell Mol Med 2021;25:10857–10868. doi: 10.1111/jcmm.16909.

42. Zhou HZ, Chen B, Li XJ, Du JJ, Zhang N, Shao YX, *et al*. MicroRNA-545-5p regulates apoptosis, migration and invasion of osteosarcoma by targeting dimethyladenosine transferase 1. Oncol Lett 2021;22:763. doi: 10.3892/ol.2021.13024.

43. Cui J, Pan G, He Q, Yin L, Guo R, Bi H. MicroRNA-545 targets ZEB2 to inhibit the development of non-small cell lung cancer by inactivating Wnt/beta-catenin pathway. Oncol Lett 2019;18:2931–2938. doi: 10.3892/ol.2019.10619.

44. Chen S, Lu S, Yao Y, Chen J, Yang G, Tu L, *et al*. Downregulation of hsa_circ_0007580 inhibits non-small cell lung cancer tumorigenesis by reducing miR-545-3p sponging. Aging (Albany NY) 2020;12:14329–14340. doi: 10.18632/aging.103472.

45. Hu C, Wang Y, Li A, Zhang J, Xue F, Zhu L. Overexpressed circ_0067934 acts as an oncogene to facilitate cervical cancer progression via the miR-545/EIF3C axis. J Cell Physiol 2019;234:9225–9232. doi: 10.1002/jcp.27601.

46. Wang HH, Ma JN, Zhan XR. Circular RNA Circ_0067934 Attenuates Ferroptosis of Thyroid Cancer Cells by miR-545-3p/SLC7A11 Signaling. Front Endocrinol (Lausanne) 2021;12:670031. doi: 10.3389/fendo.2021.670031.

47. Gao Y, Liu C, Xu X, Wang Y, Jiang Y. Circular RNA sterile alpha motif domain containing 4A contributes to cell 5-fluorouracil resistance in colorectal cancer by regulating the miR-545-3p/6-phosphofructo-2-kinase/fructose-2,6-bisphosphataseisotype 3 axis. Anti Cancer Drugs 2022;33:553–563. doi: 10.1097/CAD.0000000000001285.

48. Wang L, Wang X, Yan P, Liu Y, Jiang X. LINC00261 suppresses cisplatin resistance of esophageal squamous cell carcinoma through miR-545-3p/MT1M axis. Front Cell Dev Biol 2021;9:687788. doi: 10.3389/fcell.2021.687788.

49. Ma X, Luo J, Zhang Y, Sun D, Lin Y. LncRNA MCM3AP-AS1 upregulates CDK4 by sponging miR-545 to suppress G1 arrest in colorectal cancer. Cancer Manag Res 2020;12:8117–8124. doi: 10.2147/CMAR.S247330.

50. He M, Feng L, Qi L, Rao M, Zhu Y. Long noncoding RNASBF2-AS1 promotes gastric cancer progression via regulating miR-545/EMS1 axis. BioMed Res Int 2020;2020:6590303. doi: 10.1155/2020/6590303.

51. Liao C, Xiao W, Zhu N, Liu Z, Yang J, Wang Y, *et al*. MicroR-545 enhanced radiosensitivity via suppressing Ku70 expression in Lewis lung carcinoma xenograft model. Cancer Cell Int 2015;15:56. doi: 10.1186/s12935-015-0207-z.
